# Deletion of hepatic carbohydrate response element binding protein (ChREBP) impairs glucose homeostasis and hepatic insulin sensitivity in mice

**DOI:** 10.1016/j.molmet.2017.07.006

**Published:** 2017-07-18

**Authors:** Tara Jois, Weiyi Chen, Victor Howard, Rebecca Harvey, Kristina Youngs, Claudia Thalmann, Pradip Saha, Lawrence Chan, Michael A. Cowley, Mark W. Sleeman

**Affiliations:** 1 Department of Physiology, Monash University, Clayton, Victoria, Australia; 2 Department of Biochemistry and Molecular Biology, Monash University, Clayton, Victoria, Australia; 3 Diabetes and Endocrinology Research Center, Department of Medicine, Baylor College of Medicine, Houston, TX, USA; 4 Biomedicine Discovery Institute, Monash University, Clayton, Victoria, Australia

**Keywords:** ChREBP, Liver, Insulin sensitivity, Glucose homeostasis, Hepatic glucose production

## Abstract

**Objective:**

Carbohydrate response element binding protein (ChREBP) is a transcription factor that responds to glucose and activates genes involved in the glycolytic and lipogenic pathways. Recent studies have linked adipose ChREBP to insulin sensitivity in mice. However, while ChREBP is most highly expressed in the liver, the effect of hepatic ChREBP on insulin sensitivity remains unknown. To clarify the importance of hepatic ChREBP on glucose homeostasis, we have generated a knockout mouse model that lacks this protein specifically in the liver (Liver-ChREBP KO).

**Methods:**

Using Liver-ChREBP KO mice, we investigated whether hepatic ChREBP deletion influences insulin sensitivity, glucose homeostasis and the development of hepatic steatosis utilizing various dietary stressors. Furthermore, we determined gene expression changes in response to fasted and fed states in liver, white, and brown adipose tissues.

**Results:**

Liver-ChREBP KO mice had impaired insulin sensitivity as indicated by reduced glucose infusion to maintain euglycemia during hyperinsulinemic-euglycemic clamps on both chow (25% lower) and high-fat diet (33% lower) (p < 0.05). This corresponded with attenuated suppression of hepatic glucose production. Although Liver-ChREBP KO mice were protected against carbohydrate-induced hepatic steatosis, they displayed worsened glucose tolerance. Liver-ChREBP KO mice did not show the expected gene expression changes in liver in response to fasted and fed states. Interestingly, hepatic ChREBP deletion also resulted in gene expression changes in white and brown adipose tissues, suggesting inter-tissue communication. This included an almost complete abolition of BAT ChREBPβ induction in the fed state (0.15-fold) (p = 0.015) along with reduced lipogenic genes. In contrast, WAT showed inappropriate increases in lipogenic genes in the fasted state along with increased PEPCK1 in both fasted (3.4-fold) and fed (5.1-fold) states (p < 0.0001).

**Conclusions:**

Overall, hepatic ChREBP is protective in regards to hepatic insulin sensitivity and whole body glucose homeostasis. Hepatic ChREBP action can influence other peripheral tissues and is likely essential in coordinating the body's response to different feeding states.

## Highlights

• Liver ChREBP is protective in maintaining hepatic insulin sensitivity and regulating hepatic glucose production.
• Hepatic ChREBP deletion protects against carbohydrate-diet induced hepatic steatosis but this is not metabolically favorable.
• Deletion of hepatic ChREBP alters gene expression in adipose tissues suggesting complex inter-tissue communication.

## 1 Introduction

The coordinated control to fasting and feeding to regulate energy balance is vital in providing continual fuel for cellular processes. When food is abundant, energy must be stored for later use, and, during fasting, it must be mobilized and delivered throughout the body. Key tissues involved in these processes are the liver, muscle and adipose tissues. The reliance of various tissues in the body on glucose as a fuel substrate means blood glucose concentration in particular must be maintained. This means during periods of fasting the liver releases glucose into the bloodstream. One of the key actions of insulin in the fed state is to suppress this hepatic glucose production as well as increase uptake of glucose in muscle and adipose tissues. Impairments in the action of insulin can lead to impaired fasting glucose or glucose intolerance which are key risk factors for the development of diabetes [1]. Diabetes prevalence is increasing throughout the world and represents a serious issue to both individual and society with detrimental microvascular and macrovascular complications and significant economic disease burden [1], [2], [3], [4]. New insights into the control of insulin sensitivity and glucose homeostasis are vital for potentially uncovering new therapeutic targets and strategies.

A key transcription factor involved in coordinating the feeding response is carbohydrate response element binding protein (ChREBP). ChREBP is a transcription factor that regulates numerous genes in response to changes in cellular glucose concentration. Pathway analysis of ChREBP target genes has revealed roles for ChREBP in lipid and carbohydrate metabolism, cell motility, and insulin signaling [5], [6]. Furthermore, human association studies have linked ChREBP with insulin sensitivity, plasma triglycerides, and coronary artery disease [7], [8], [9], [10], [11], [12], [13], [14], [15], [16]. Increased expression of ChREBP in adipose tissue is linked to improved insulin sensitivity in humans [7], [10], [13]. Recently, investigations into the function of ChREBP in adipose tissue revealed a novel class of lipids, termed fatty acid esters of hydroxy fatty acids (FAHFAs), that have both anti-inflammatory and insulin-sensitizing properties [16]. In contrast, association studies have linked increased ChREBP expression in liver to hepatic steatosis and insulin resistance [7], [13]. As ChREBP regulates genes involved in *de novo* lipogenesis, the association found with hepatic steatosis is unsurprising. Selective over-expression of ChREBP in the liver of mice worsens hepatic steatosis [17], and inhibition of hepatic ChREBP in ob/ob mice reduces it [18]. However, the relationship of hepatic ChREBP with insulin sensitivity is less clear. Inhibition of hepatic ChREBP in ob/ob mice improves insulin sensitivity and glucose tolerance; yet, mice with hepatic over-expression of ChREBP do not have impairments in insulin signaling and in fact have improved insulin sensitivity and glucose tolerance when fed a high-fat diet [17], [18]. Clearly, ChREBP in liver, not only in adipose tissue, may play an important role in lipid metabolism, glucose homeostasis, and insulin sensitivity and therefore metabolic disease in humans.

We sought to clarify the role of hepatic ChREBP in glucose homeostasis and insulin sensitivity using a novel mouse model of hepatic ChREBP deletion. We describe the physiological consequences of specific liver ChREBP deletion in mice and have identified an important role for hepatic ChREBP in whole body glucose homeostasis and hepatic insulin sensitivity.

## 2 Methods

### 2.1 Animals and husbandry

Liver-specific ChREBP KO mice were created by breeding floxed mice (ChREBP fl/fl in which loxP sites surround a critical exon in the mlxipl gene) to a line expressing the Cre recombinase specifically in liver (Alb-cre). The resultant homozygous progeny (Liver-ChREBP KO) do not show expression of ChREBP in the liver. Group housed animals were kept on a 12-h light–dark cycle with free access to food and water, with body weights recorded weekly. All conditions and experiments were reviewed and approved by Monash University Animal Ethics committee. Experimental diets were generated by Specialty Feeds (Glen Forrest, Western Australia). High fat diet (SF04-001, 45% calories from lipids) was based on Research Diets D12451. High carbohydrate diet (SF13-067, 70% calories from carbohydrates) was created using SF04-001 as a template with similar vitamin and mineral composition. Detailed comparison of the high-fat and high-carbohydrate diets is given in Supplemental Table 1. A chow diet (#12145) was used as a control chow in all experiments.

### 2.2 Body composition

Body composition was assessed by dual-energy X-ray absorptiometry (DEXA) (Piximus, Lunar).

### 2.3 Indirect calorimetry

Mice were individually housed in metabolic chambers (CLAMS, Columbus Instruments) in order to assess metabolic activity. Mice were acclimated for 48 h before data were collected. Mice had free access to food and water for baseline recordings, or were subjected to a fasting-refeeding protocol in which food was removed for 24 h at the beginning of the night period, and recordings were taken during fasting, refeeding, and recovery. Oxygen consumption, carbon dioxide production, and ambulatory activity were measured, and RER and energy expenditure were calculated.

### 2.4 Glucose tolerance

To assess glucose tolerance, 18-hour fasted animals were dosed with a 3 g/kg glucose solution P.O. and blood glucose was measured at 0, 30, 60, and 120 min via tail blood using a handheld glucometer (ACCU-CHEK, Roche Diagnostics).

### 2.5 Hyperinsulinemic-euglycemic clamps and glucose uptake

Mice were subjected to jugular vein catheterization 5 days prior to hyperinsulinemic euglycemic clamp studies. Mice were anesthetized with isoflurane (2–3% in oxygen) while an indwelling silastic catheter was inserted into the right internal jugular vein and exteriorized through the back of the neck. The catheters were kept patent with heparin sodium (1 IU/ml, Pfizer) and sealed with a stainless steel plug. Mice were allowed 4–5 days postsurgical recovery. Food pellets were placed at the bottom of the cage to facilitate recovery. Body weight was recorded daily, and mice that had less than 5% weight loss were subsequently studied.

Hyperinsulinemic euglycemic clamps followed by 2-deoxyglucose uptake were performed on 6 h fasted, conscious, and unrestrained mice as described previously [19]. Insulin infusion rate for chow fed mice was 2 mU/kg/min, and for high-fat diet fed animals was 4 mU/kg/min. Soleus, extensor digitorum longus, gastrocnemius, tibialis anterior, kidney, liver, WAT (visceral, perigonadal and subcutaneous), BAT, and heart were assessed for 2[14C]DG radioactivity.

### 2.6 Liver histology

For lipid staining, livers were frozen in liquid nitrogen-cooled isopentane then embedded in OCT (4583, Tissue-Tek). Frozen blocks were sectioned at 10 μm, mounted and dried, and stained with Oil Red O or Hematoxylin & Eosin. For trichrome staining and periodic acid-Schiff (PAS) staining, livers were preserved in 10% formalin (w/v) for 24–48 h followed by embedding in paraffin, sectioning at 4 μm and staining with Masson's trichrome, PAS, or Hematoxylin & Eosin.

### 2.7 Liver glycogen

To measure liver glycogen, 200 mg frozen liver was homogenized in 20 mM Tris HCL with 0.1% Triton-X100. Samples were spun at 2700 × g at 4 °C for 20 min, and the supernatant was used for protein BCA assay and glycogen assay. For glycogen assay, samples were precipitated overnight at 4 °C with ethanol, reconstituted with 0.04 M Na acetate buffer followed by incubation in Na acetate buffer either with or without amyloglucosidase for 1 h at 37 °C. The hydrolyzed free glucose concentration was measured with Glucose (HK) Assay Kit (GAHK-20, Sigma).

### 2.8 Liver lipogenesis and fatty acid oxidation

For all experiments, a modified Kreb's-Henseleit buffer was gassed for 40 min with 95% O2/5% CO2. Glucose (5 mM) and fatty acid-free BSA (4%) was added to the buffer immediately before experiments. All experiments were conducted in a shaking water bath at 30 °C.

For hepatic lipogenesis d-[3-3H]Glucose (TRK239; Amersham, Rydalmere, New South Wales, Australia) was added to the buffer to give a final concentration of 0.5 μCi/ml. Liver was sliced into 1–2 mm explants and incubated for 2 h, and the medium was removed. The tissue was washed in PBS and then homogenized in 1 ml PBS. The lipids were extracted in 2:1 chloroform-methanol, a 1-ml aliquot of the organic phase was removed, scintillation fluid was added, and radioactivity was counted in a liquid scintillation analyzer.

For analysis of oxidation all experiments, liver was sliced into 1–2 mm and explants were placed in warmed (30 °C) Krebs-Henseleit buffer pH 7.4 containing 2 mm pyruvate, 4% fatty acid-free bovine serum albumin (Bovogen, VIC, Australia) and 1 mm palmitic acid (Sigma, St Louis, MO, USA). After an initial incubation of 20 min, the incubation buffer was replaced with the same buffer described above supplemented with 0.5 μCi ml^−1^ of [1-14C]palmitate (Amersham BioSciences, Little Chalfont, UK).

### 2.9 Blood chemistry

Blood samples were obtained by retro-orbital bleeds under isoflurane anesthesia. Plasma glucagon, ghrelin, GIP, GLP-1, insulin, leptin, PAI-1, and resistin were determined by multiplexed bead assay (Bio-Plex Pro mouse diabetes assay, Bio-Rad) using the MAGPIX instrument (Luminex).

Serum triglyceride and fatty acid levels were determined by colorimetric reaction (432-40201 & 279-75401, WAKO).

### 2.10 Gene expression

RNA was extracted from liquid nitrogen flash-frozen samples and purified using a column-based RNeasy 96 QIAcube HT Kit (74171, Qiagen). Extracted RNA was quantified and checked for purity using the QIAxpert system (Qiagen). cDNA was generated from 1 μg RNA using iScript Advanced cDNA Synthesis Kit for RT-qPCR (170-8843; Bio-Rad). qPCR was run for ChREBP-α (F; CGACACTCACCCACCTCTTC, R; TTGTTCAGCCGGATCTTGTC), ChREBP-β (F; TCTGCAGATCGCGTGGAG, R; CTTGTCCCGGCATAGCAAC) and 36B4 (F; GCGACCTGGAAGTCCAACTAC, R; ATCTGCTGCATCTGCTTGG) (reference gene) using custom oligonucleotides (MicroMon, Monash University) and SYBR Green PCR Master Mix (4309155, Life Technologies). Amplifications were performed using an Applied Biosystems 7900HT instrument, followed by a melt curve analysis.

Additionally, qPCR was performed for other target genes using Taqman assays (Table 1) and the Fluidigm Biomark HD system at MHTP Medical Genomics Facility. Data were analyzed using Fluidigm Real-Time PCR analysis software (V4.1.1).

**Table 1:** Gene expression targets for Liver-ChREBP KO mice.

Target	Life tech code
SREBP1	Mm00550338_m1
MondoA	Mm01202115_m1
RPLP0	Mm00725448_s1
ACC	Mm01304257_m1
SIRT1	Mm00490758_m1
SCD1	Mm00772290_m1
PEPCK	Mm01247058_m1
HPRT	Mm01545399_m1
GCGR	Mm00433546_m1
TXNIP	Mm01265659_g1
FAS	Mm00662319_m1
TBP	Mm00446971_m1
HNF4A	Mm01247712_m1
IRF5	Mm00496477_m1
LXRα	Mm00443451_m1
IL1β	Mm00434228_m1
TRβ	Mm00437044_m1
FOXO1	Mm00490672_m1
GLUT4	Mm01245502_m1
PPARα	Mm00440939_m1
FGF21	Mm00840165_g1
GLUT2	Mm00446229_m1
TNFα	Mm00443258_m1
PAI-1	Mm00435860_m1

### 2.11 Statistical analysis

All data are presented as mean ± SEM, with a statistically significant difference defined as p < 0.05. For the analysis of calorimetry data, general estimating equation models were used to provide population average effects to take into account the correlation across time. Adjustment was undertaken to assess the effects of individual total body weight, lean and fat mass. All graphs and statistical analyses were completed using StataCorp 2015 software (*Stata Statistical Software*: *Release 14*, College Station, TX; StataCorp LP). Additional graphs and statistical analyses were completed using GraphPad Prism (GraphPad Prism 6.0 for Mac OS X, GraphPad Software, Inc., San Diego, CA) as indicated.

## 3 Results

### 3.1 Liver-ChREBP KO mice display impaired glucose tolerance and hepatic insulin resistance

Body composition and metabolic activity were assessed via DEXA scanning and CLAMS metabolic cages in Liver-ChREBP KO mice and WT or Fl/Fl control mice fed either a chow or high-fat diet. Liver-ChREBP KO mice trended towards a lower body weight, had no significant difference in lean body mass, and had reduced total and percentage body fat compared to control mice (Supplemental Figure 6 A–C; p < 0.01). Although Liver-ChREBP KO mice started with less body fat, after eight weeks on high-fat diet, there was no difference in body weight, lean mass, total fat mass, or percentage fat with control mice (Supplemental Figure 6D). There were no significant differences in bone mineral density or bone mineral content with any diet (data not shown). Liver-ChREBP KO mice had increased absolute oxygen consumption and carbon dioxide production in the day and night periods (p < 0.05), as well as increased energy expenditure at night (p < 0.05) (Supplemental Figures 1–6). The increased energy expenditure in Liver-ChREBP KO mice was present despite no significant difference in activity levels. The increased RER suggests that these mice have increased carbohydrate oxidation compared to control mice and may reflect a change in substrate preference for energy production. In order to further investigate the alterations in RER, mice underwent a fasting-refeeding protocol in which mice were placed into metabolic cages for five days and subjected to a 24-hour fast from 7pm to 7pm in the middle of this period. As expected the RER greatly reduced during the fast as mice switch to oxidizing more fats (Supplemental Figure 6E). At the beginning of each night period, Liver-ChREBP KO mice display an increased RER, and this effect is greatly exaggerated after refeeding following the 24-hour fast (Supplemental Figure 6E).

**Figure 1:**
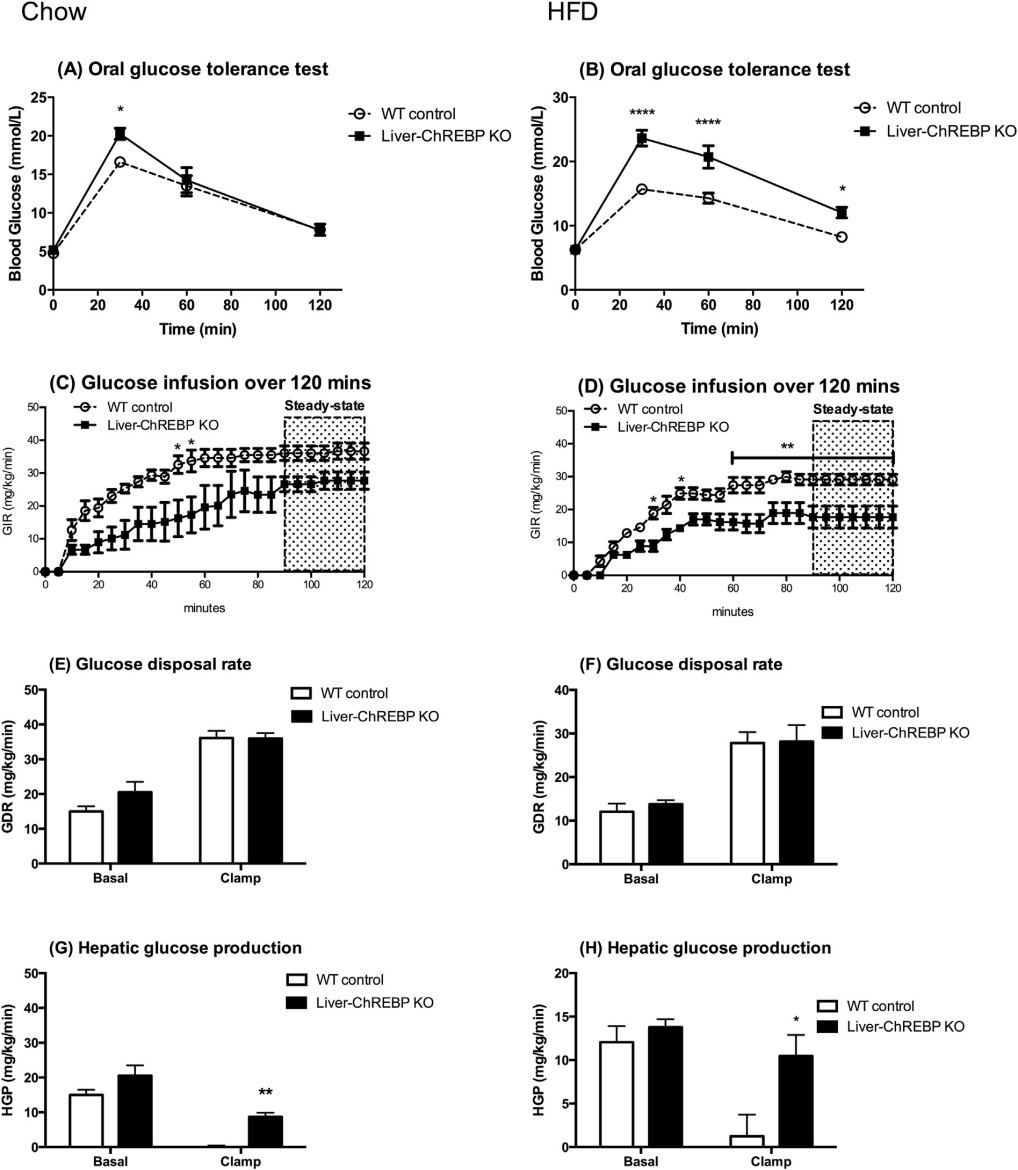
**Liver-ChREBP KO mice display impaired glucose tolerance and hepatic insulin resistance.** Oral glucose tolerance test (oGTT) in Liver-ChREBP KO and WT mice (n= 5–7 per group) on chow (A) or HFD (B). Glucose infusion rate (GIR) over 120 min, GIR, Glucose disposal rate (GDR) and Hepatic glucose production (HGP) during a hyperinsulinemic-euglycemic clamp (n=4 per group) on chow (C,E,G) or HFD (D,F,H). Results expressed as mean ± SEM. Statistical analysis by two-way ANOVA followed by Tukey’s post-hoc test (*: p<0.05, **: p<0.01, ****: p<0.0001).

In order to examine the effect of hepatic ChREBP deletion on the ability to handle a glucose load, a glucose tolerance test (GTT) was performed. Liver-ChREBP KO mice showed elevated blood glucose 30 min post receiving a glucose load orally, suggesting an impairment in the ability to clear glucose (Figure 1A) (p < 0.05). This effect was exaggerated when mice were placed on a high-fat diet for 12 weeks, where Liver-ChREBP KO mice showed greatly impaired glucose tolerance compared to control mice (Figure 1B) (p < 0.0001). This was despite no differences in body composition in high-fat diet fed mice and suggests that Liver-ChREBP KO mice are more susceptible to diet-induced glucose intolerance. To assess whether the impairments in glucose tolerance were due to problems in insulin sensitivity, hyperinsulinemic-euglycemic clamps were performed on Liver-ChREBP KO and control mice fed either a chow or high-fat diet. Liver-ChREBP KO mice had a reduced glucose infusion rate to maintain euglycemia during clamps on both chow (25% lower) and high-fat diet (33% lower) (Figure 1C,D) (p < 0.05). This is suggestive of impaired systemic insulin sensitivity. Interestingly, there were no significant differences in glucose disposal rate on either diet, suggesting muscle insulin sensitivity was not affected (Figure 1E,F). In support of this, there were no significant differences in glucose uptake in skeletal muscle (Supplemental Figure 7A). Strikingly, the ability of insulin to suppress hepatic glucose production was greatly impaired in both chow (58% reduction compared to 100%) and high-fat (24% reduction compared to 90%) conditions in Liver-ChREBP KO mice, suggesting hepatic insulin resistance and explaining the difference in glucose infusion rate (Figure 1G,H) (p < 0.05). The blood glucose and insulin levels prior and during clamps on chow or HFD are given in Supplemental Figure 7.

### 3.2 Liver-ChREBP KO mice are protected from high-carbohydrate diet induced hepatic steatosis

**Figure 2:**
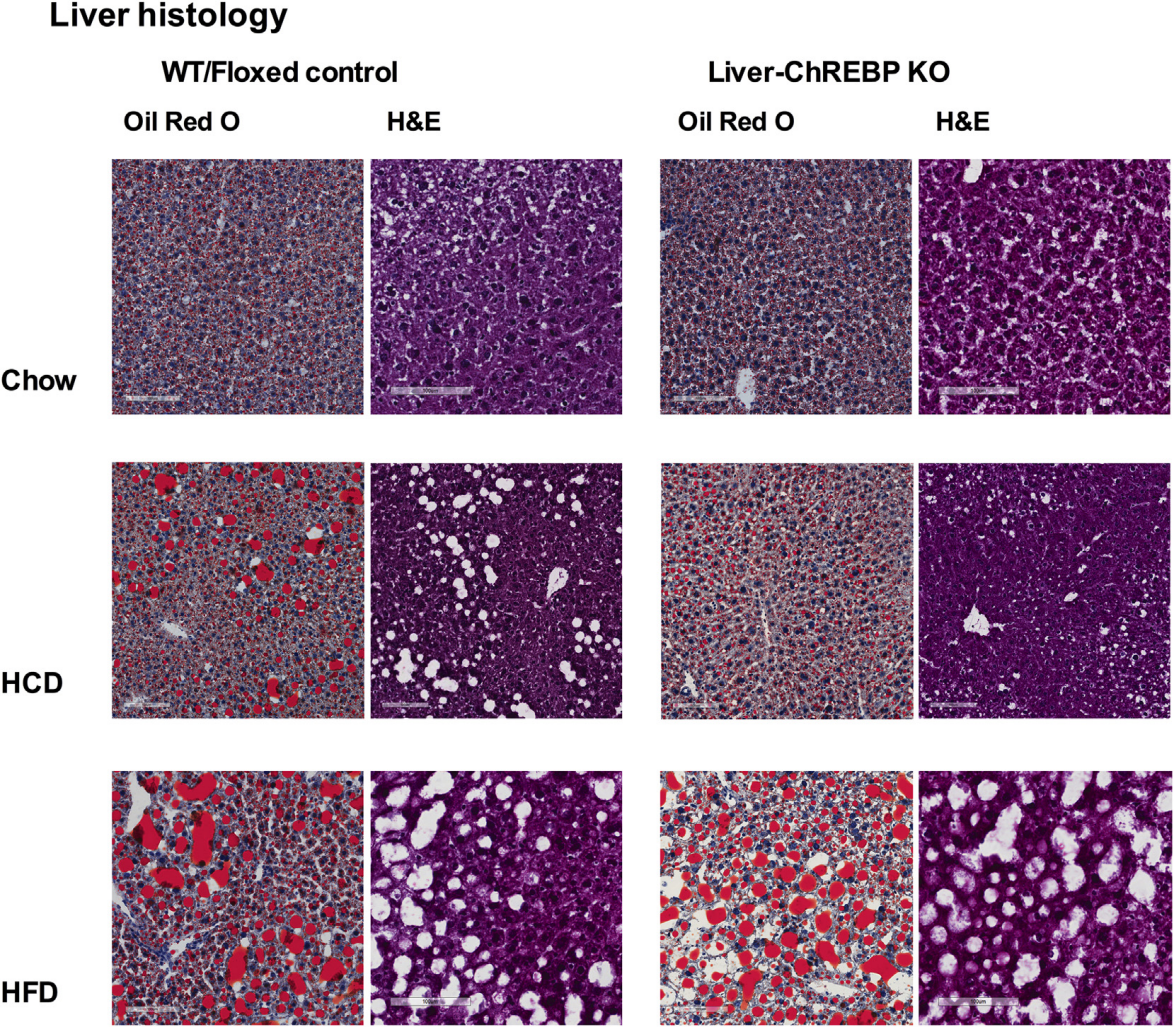
**Liver histology in Liver-ChREBP KO mice.** Liver histology showing representative Oil Red O and H&E stained liver sections from Liver-ChREBP KO and WT or Fl/Fl mice fed either a chow, HCD or HFD for 12 weeks. Magnification = 20×.

ChREBP is a lipogenic transcription factor that is known to be involved in the development of hepatic steatosis. In order to determine the effect of hepatic ChREBP deletion on hepatic steatosis development, livers were histologically assessed for fat deposition via Oil Red O staining in Liver-ChREBP KO and control mice fed either a normal chow, a high-fat diet or a high-calorie high-carbohydrate diet. A representative image from Liver-ChREBP KO and wildtype or Fl/Fl control mice fed a chow, high-fat or high-carbohydrate diet for 12 weeks is displayed in Figure 2. On a normal chow diet there was minimal fat deposition in livers of mice of either genotype. When fed a high-fat diet both control and Liver-ChREBP KO mice displayed hepatic steatosis with increased fat deposition evident. However, when fed a high-carbohydrate diet, Liver-ChREBP KO mice showed greatly reduced hepatic lipid deposition when compared to control mice (Figure 2). This suggests that ChREBP plays a role in the development of hepatic steatosis in response to high carbohydrate diets. Interestingly, this reduction in hepatic steatosis did not improve metabolic health in these mice. In fact, further physiological assessment of high-carbohydrate diet fed mice found Liver-ChREBP KO mice had worsened glucose tolerance compared to control mice despite reduced body weight and reduced body fat (Supplemental Figure 9D). Interestingly, Liver-ChREBP KO mice display reduced adipocyte size in white adipose tissue when compared to WT control mice (Supplemental Figure 10). This reduced adipocyte size fits with the reduction in fat mass seen in Liver-ChREBP mice and suggests there may be alterations in fatty acid flux in this tissue.

### 3.3 Liver-ChREBP KO mice have reduced hepatic fatty acid oxidation and increased plasma inflammatory markers

**Figure 3:**
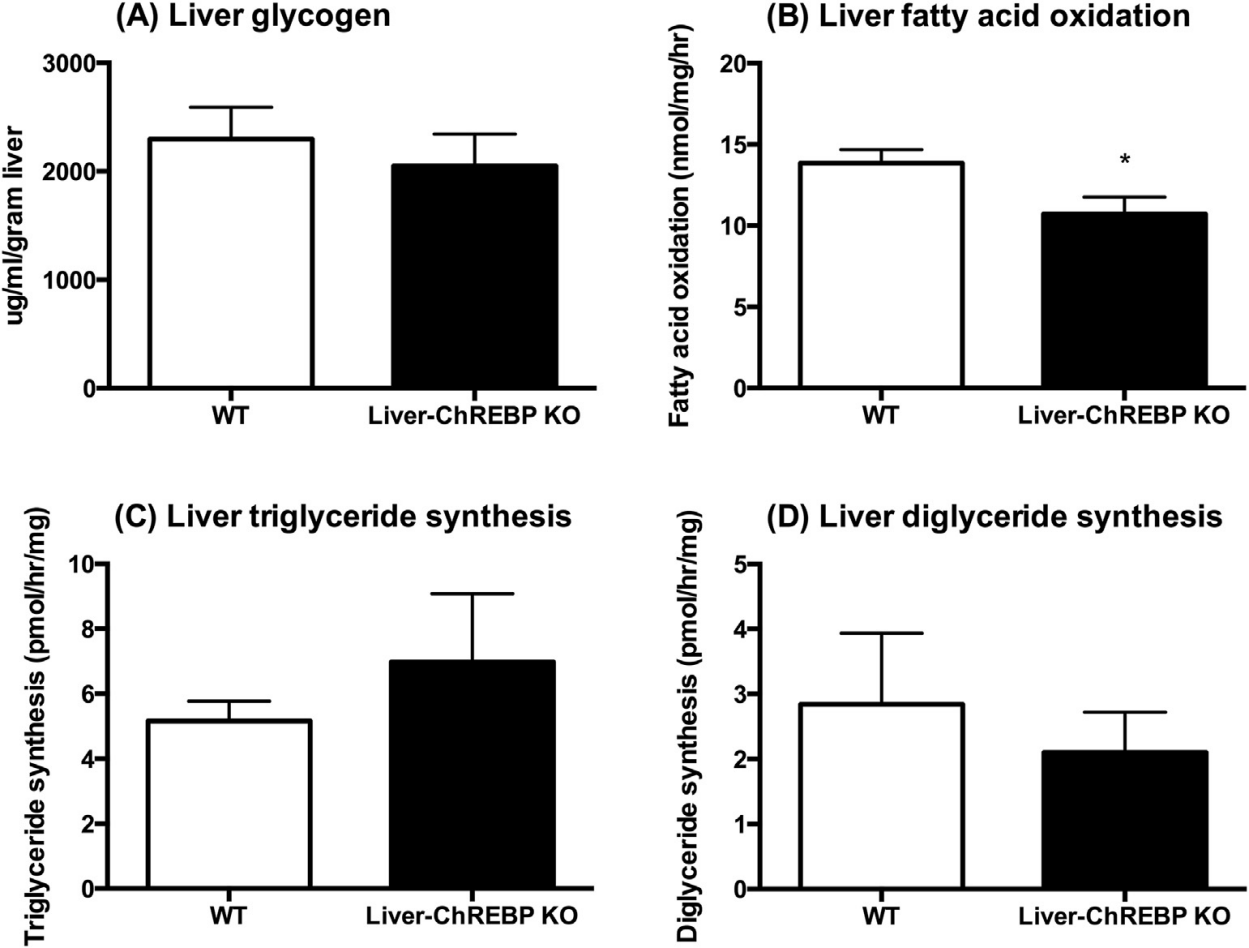
**Liver metabolism in chow-fed Liver-ChREBP KO mice.** A. Liver glycogen content (n=4 per group). B. Fatty acid oxidation rate in isolated liver ex vivo (n=6 per group). C. Lipogenesis of TAG and D. DAG in isolated liver ex vivo (n=6 per group). Results expressed as mean ± SEM. Statistical analysis by unpaired t-test between each genotype (*: p<0.05, **: p<0.01, ***: p<0.001, ****: p<0.0001).

**Figure 4:**
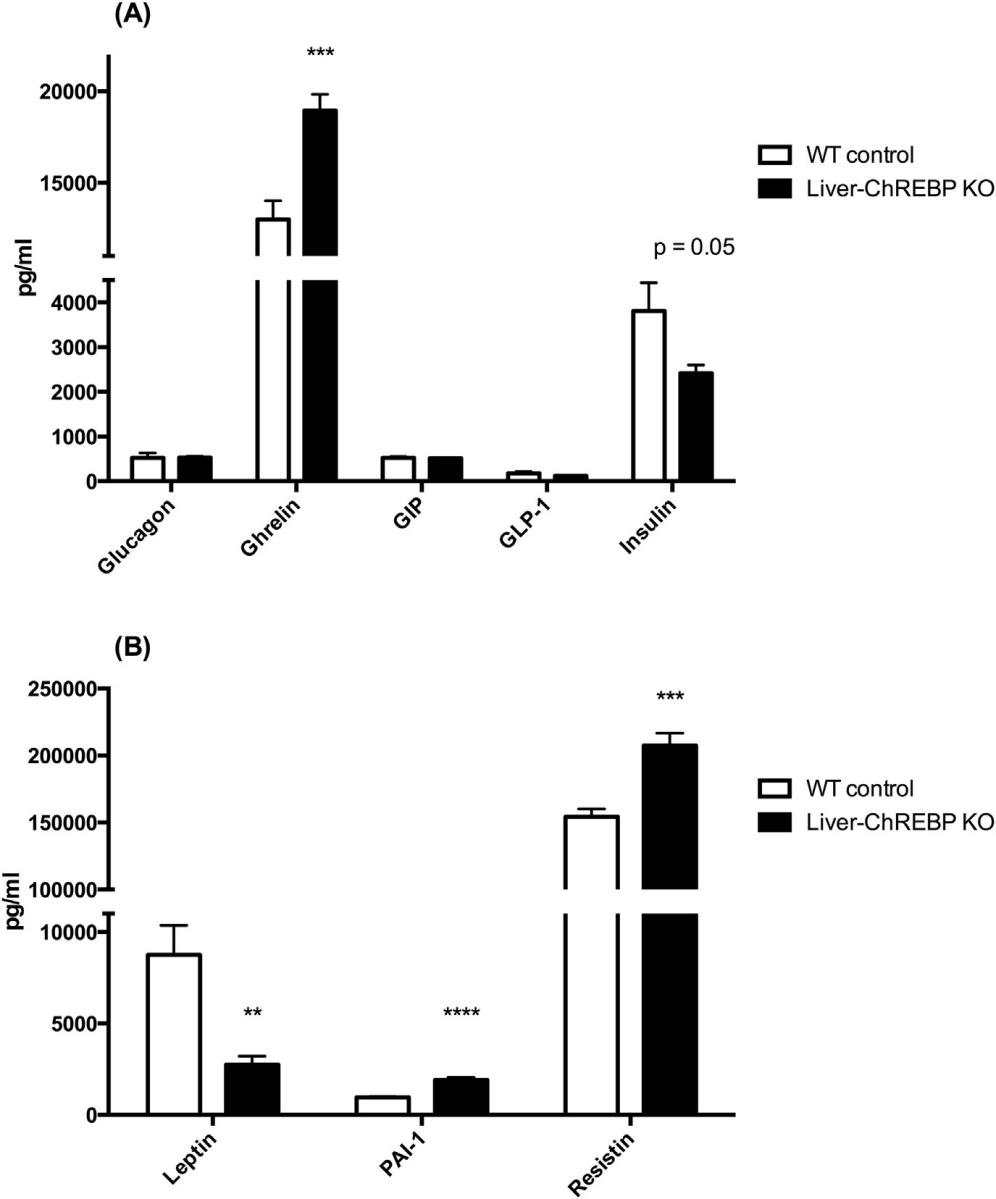
**Plasma metabolic biomarkers in chow-fed Liver-ChREBP KO mice.** A. and B. Concentrations of metabolic biomarkers in plasma of Liver-ChREBP KO and WT mice (n=9 per group). Results expressed as mean ± SEM. Statistical analysis by unpaired t-test between each genotype (**: p<0.01, ***: p<0.001, ****: p<0.0001).

In order to assess the effect of hepatic ChREBP deletion on liver metabolic function, glycogen content, fatty acid oxidation, and lipogenesis were measured in chow-fed mice. There was no significant difference in liver glycogen content between genotypes, suggesting no impairments with glycogen synthesis and storage in Liver-ChREBP KO mice (Figure 3A). This was confirmed with PAS staining in liver sections (Supplemental Figure 10). Liver fatty acid oxidation was reduced by 23% in Liver-ChREBP KO mice (Figure 3B) (p < 0.05), which supports a potential change in substrate preference as indicated by the increase in RER seen in these mice. However, lipogenesis was unaffected in Liver-ChREBP KO mice, suggesting compensation can occur for lack of hepatic ChREBP (Figure 3C,D). Assessment of plasma metabolic biomarkers in chow-fed Liver-ChREBP KO mice revealed a 69% reduction in leptin, which fits with the reduction in body fat in these mice (Figure 4B) (p < 0.01). Liver-ChREBP KO mice also showed a 46% increase in plasma ghrelin, which is likely an inappropriate increase as blood was taken in the fed state when ghrelin levels should be low (Figure 4A) (p < 0.001). Interestingly, Liver-ChREBP KO mice had significantly elevated levels of the inflammatory markers plasminogen activator inhibitor-1 (PAI-1) (103% increase) and resistin (35% increase), which is suggestive of a heightened inflammatory profile (Figure 4B) (p < 0.001).

### 3.4 Selective deletion of ChREBP in liver alters ChREBP expression in brown adipose tissue

**Figure 5:**
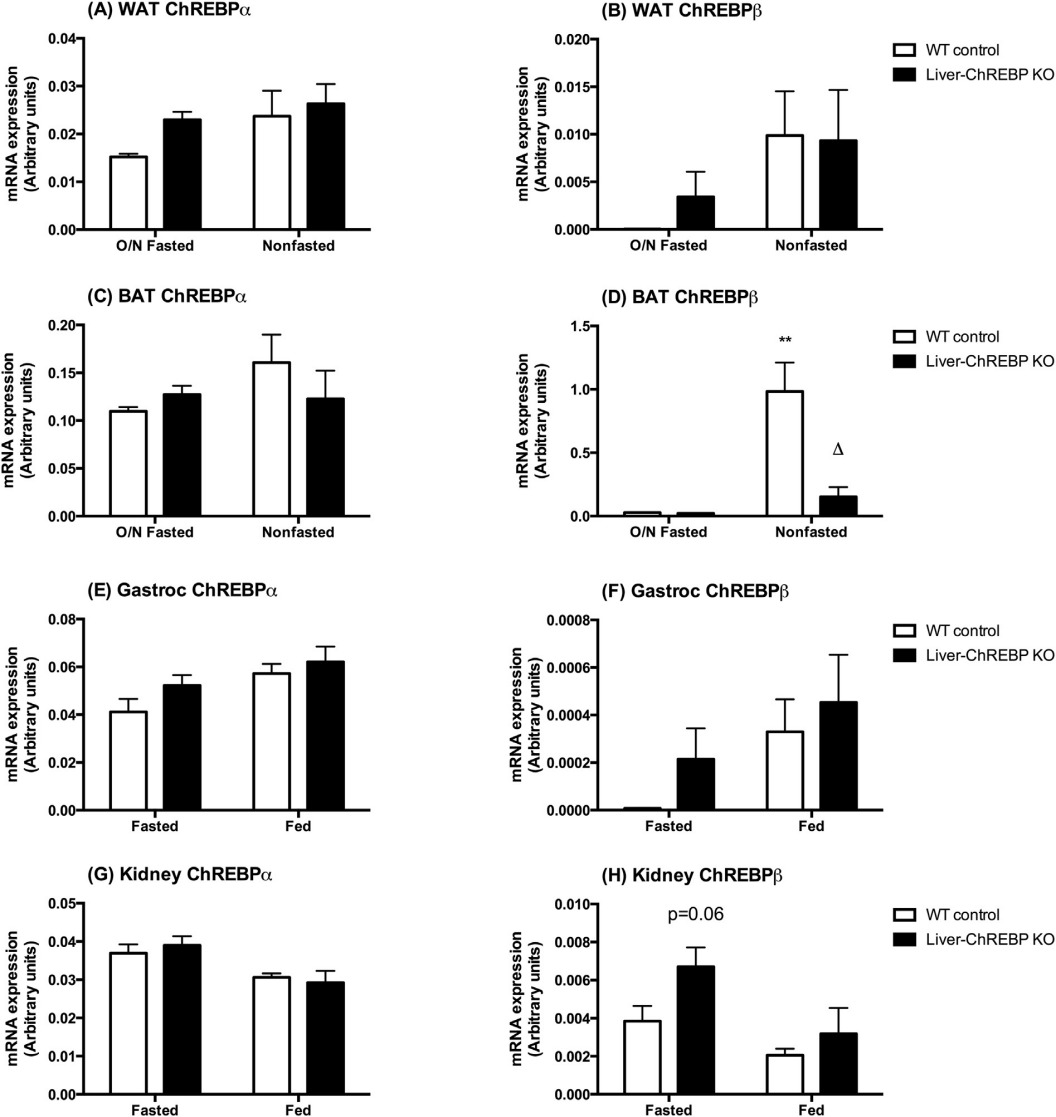
**ChREBP expression in Liver-ChREBP KO mice.** ChREBPα gene expression in A. WAT, C. BAT, E. Gastrocnemius muscle and G. Kidney of Liver-ChREBP KO and WT mice after overnight fasting (fasted), or without fasting (fed). ChREBPβ gene expression in B. WAT, D. BAT, F. Gastrocnemius muscle and H. Kidney of Liver-ChREBP KO and WT mice after overnight fasting (fasted), or without fasting (fed). Results were normalized to expression of the housekeeping gene 36B4, and expressed in arbitrary units using the 2^(–δCt) formula. Results are expressed as mean ± SEM. Statistical analysis was by two-way ANOVA followed by Tukey’s post-hoc test. * denotes significance between genotypes (within a fasting state), δ denotes significance between fasting states (within a genotype) (δ: p<0.05, **: p<0.01).

Liver-ChREBP KO mice were confirmed to have a selective ChREBP deletion in liver, with expression still evident in other tissues (Supplemental Figure 11). As tissue cross-talk is an important factor in the development of metabolic disease, the influence of hepatic ChREBP deletion on the expression of ChREBP in other tissues was investigated. Mice were either fasted overnight or left in the fed state and tissues were collected for gene expression analysis. There were no significant differences in ChREBPα expression in WAT, BAT, gastrocnemius, or kidney (Figure 5). Interestingly, there were also no significant differences in ChREBPα expression between fasting states in any tissue, supporting the notion that it is the ChREBPβ isoform that is more transcriptionally regulated in response to feeding. Correspondingly, in control and Liver-ChREBP KO mice ChREBPβ expression increased in WAT in the fed compared to the fasted state, although this did not reach statistical significance (Figure 5B). In BAT, control mice had a robust increase in ChREBPβ expression in the fed state (Figure 5D) (p < 0.01). In contrast, this upregulation in the fed state was almost abolished (0.15-fold of normal response) in Liver-ChREBP KO mice (Figure 5D) (p < 0.05). There were no significant differences in ChREBPβ expression in gastrocnemius and a trend towards increased kidney ChREBPβ expression in the fasted state in Liver-ChREBP KO mice (Figure 5F,H).

### 3.5 Liver-ChREBP KO mice have altered expression of lipogenic genes in liver and adipose tissues

ChREBP is a transcription factor that regulates various genes involved in glycolysis, lipogenesis, and glucose metabolism. In order to look at the effect of hepatic ChREBP deletion on the mRNA expression of these genes, Liver-ChREBP KO and control mice were either fasted overnight or left in the fed state, and various tissues were collected for gene expression analysis. In addition to ChREBP target genes, other lipogenic transcription factors were measured to assess any potential compensation occurring due to hepatic ChREBP deletion. Furthermore, various genes can regulate ChREBP or are involved in feedback pathways with ChREBP, and these genes were also measured. Finally, as Liver-ChREBP KO mice seem to be in a heightened inflammatory state, as demonstrated by their increased plasma resistin and PAI-1, expression of various inflammatory markers was also measured.

**Figure 6:**
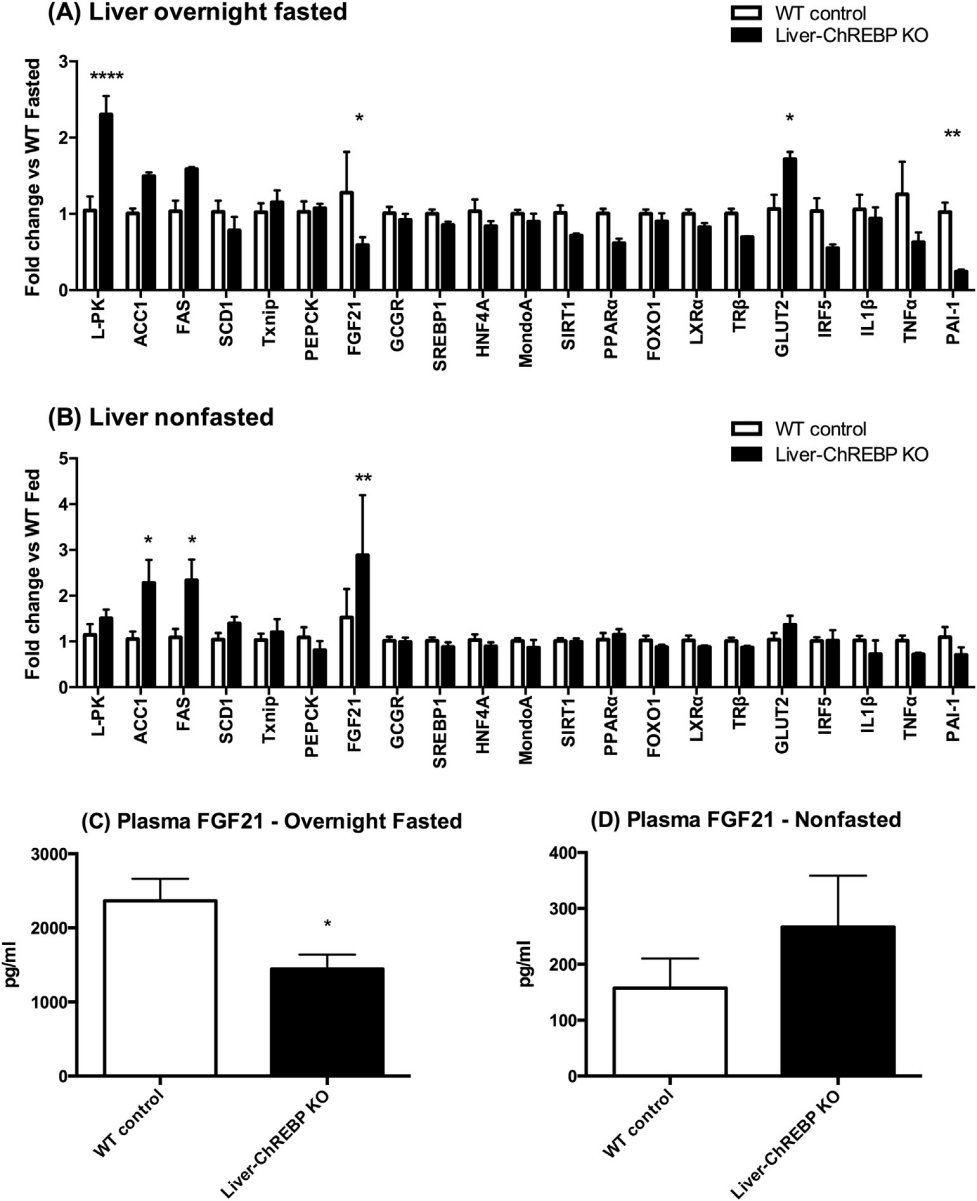
**Gene expression in liver and plasma FGF21 of chow-fed Liver-ChREBP KO mice.** (A) Gene expression in liver of Liver-ChREBP KO and WT control mice after overnight fasting, or (B) without fasting. Results were normalized to expression of one of 3 housekeeping genes; 36B4, Hprt or Pgk1; and then shown as fold change versus either the WT fasted group (A), or the WT non-fasted group (B). (C) Plasma FGF21 of Liver-ChREBP KO and WT control mice after overnight fasting, or (D) without fasting. Results are expressed as mean ± SEM. Statistical analysis was by two-way ANOVA followed by Tukey’s post-hoc test (A, B), or by unpaired t-test (C). (*: p<0.05, **: p<0.01, ****: p<0.0001).

Liver-ChREBP KO mice had various gene expression changes in the liver. In the fasted state, Liver-ChREBP KO mice had elevated liver type pyruvate kinase (L-PK) (2.3-fold) (p < 0.0001) as well a trend towards increased acetyl-coA carboxylase 1 (ACC1) (1.5-fold) and fatty acid synthase (FAS) (1.6-fold) (Figure 6A). In the non-fasted state there was no significant difference in L-PK expression, but Liver-ChREBP KO mice had increased ACC1 and FAS expression (2.3-fold) (Figure 6B) (p < 0.05). These genes are only supposed to be upregulated in the fed state, and the increases seen in both the fasted and fed states in the Liver-ChREBP KO mice suggest dysregulation of the response to glucose. Clearly, there is compensation occurring that allows upregulation of ChREBP target genes despite hepatic ChREBP deletion, but it seems this compensation cannot respond to glucose appropriately, resulting in activation of target gene expression regardless of fasting state. In support of this, Liver-ChREBP KO mice have greatly reduced expression of lipogenic genes when fed a high-fat diet (Supplemental Figure 12). This suggests that adequate compensation by other transcriptional regulators can no longer occur in the state of dietary overload. GLUT2 expression was increased in the fasted state in Liver-ChREBP KO mice (1.7-fold) (Figure 6A) (p < 0.05). Interestingly, FGF21 expression was decreased in the fasted state (0.6-fold) but increased in the fed state (2.9-fold) in Liver-ChREBP KO mice (Figure 6A/B) (p < 0.05). These hepatic gene expression changes corresponded to changes in plasma FGF21, where Liver-ChREBP KO mice had a trend towards increased FGF21 in the fed state and a significant decrease in FGF21 levels in the fasted state (Figure 6C,D) (p < 0.05). Again, when fed a high-fat diet, Liver-ChREBP KO mice in the fed state show an opposite relationship where they have significantly reduced FGF21; however, there was no detectable difference in serum FGF21 levels (Supplemental Figure 12B and C). Unexpectedly, there was a decrease in PAI-1 expression in the fasted state (0.3-fold) (Figure 6A) (p < 0.01). There were no significant differences in any of the ChREBP regulators or other genes measured.

**Figure 7:**
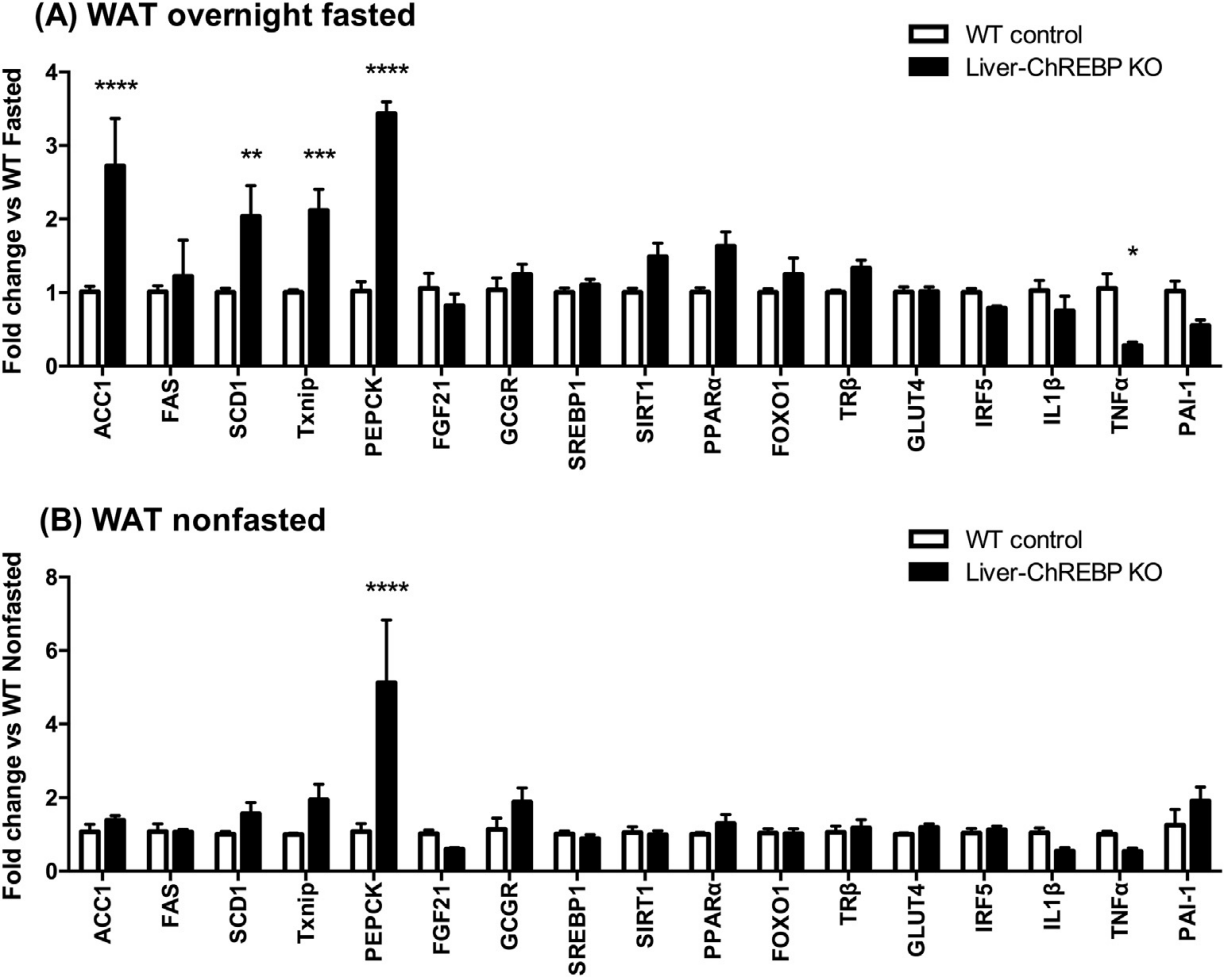
**Gene expression in white adipose tissue of chow-fed Liver-ChREBP KO mice**. (A) Gene expression in WAT of Liver-ChREBP KO and WT control mice after overnight fasting, or (B) without fasting. Results were normalized to expression of one of 3 housekeeping genes; 36B4, Hprt or Pgk1; and then shown as fold change versus either the WT fasted group (A), or the WT non-fasted group (B). Results are expressed as mean ± SEM. Statistical analysis was by two-way ANOVA followed by Tukey’s post-hoc test. (*: p<0.05, **: p<0.01, ***: p<0.001, ****: p<0.0001).

The gene expression changes in WAT show inappropriate upregulation of lipogenic genes in the fasting state in Liver-ChREBP KO mice. Expression of ACC1 (2.7-fold), SCD1 (2.0-fold) and thioredoxin interacting protein (TXNIP) (2.1-fold) are all increased in fasted Liver-ChREBP KO mice when compared to control mice (Figure 7A) (p < 0.01). In response to a high-fat diet both WT and Liver-ChREBP KO mice have the expected reduction in lipogenic gene expression (Supplemental Figure 13A). Interestingly, phosphoenolpyruvate carboxykinase (PEPCK) expression is profoundly increased in WAT of Liver-ChREBP KO mice in both fed (5.1-fold) and fasted (3.4-fold) states (Figure 7A,B) (p < 0.0001). In WAT, PEPCK is responsible for glyceroneogenesis and plays an important role in fatty acid cycling in adipocytes. Strikingly, this increase in PEPCK is absent with high-fat diet feeding (Supplemental Figure 13B). Unexpectedly, the expression of inflammatory marker tumor necrosis factor alpha (TNFα) was decreased in Liver-ChREBP KO mice in the fasted state (0.3-fold) (Figure 7A) (p < 0.05). There were no other significant changes between genotypes in other genes measured.

**Figure 8:**
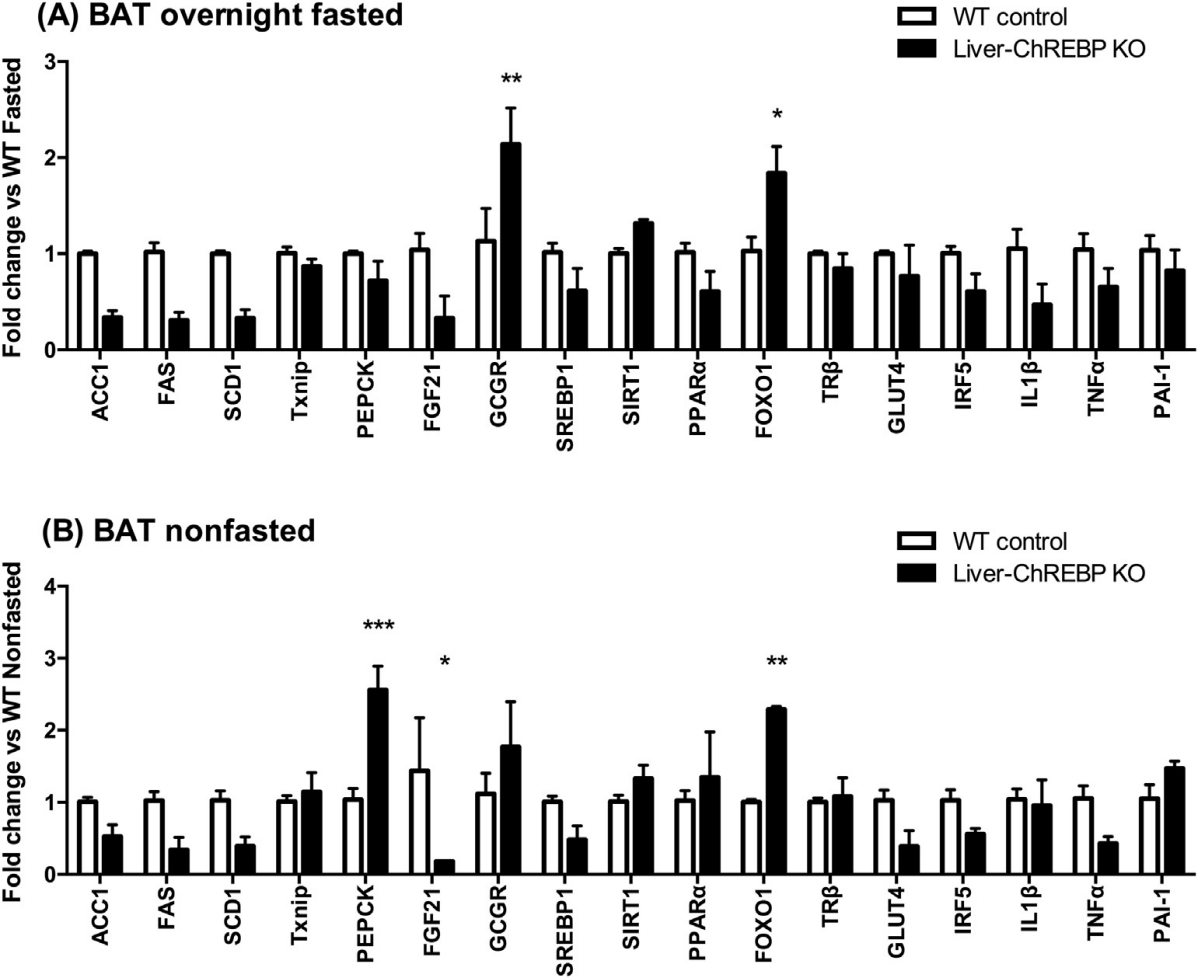
**Gene expression in brown adipose tissue of chow-fed Liver-ChREBP KO mice**. (A) Gene expression in BAT of Liver-ChREBP KO and WT control mice after overnight fasting, or (B) without fasting. Results were normalized to expression of one of 3 housekeeping genes; 36B4, Hprt or Pgk1; and then shown as fold change versus either the WT fasted group (A), or the WT non-fasted group (B). Results are expressed as mean ± SEM. Statistical analysis was by two-way ANOVA followed by Tukey’s post-hoc test. (*: p<0.05, **: p<0.01, ***: p<0.001).

The gene expression changes seen in BAT were greatly different to those seen in WAT. In the fasted state, there was a trend towards a decrease in the ChREBP target genes ACC1, FAS, SCD1, and FGF21 in Liver-ChREBP KO mice (0.3-fold) (Figure 8A). This reduction in lipogenic genes was no longer present when mice were fed a high-fat diet (Supplemental Figure 14A). There was also a significant reduction in FGF21 expression in Liver-ChREBP KO mice in the fed state (0.2-fold) (Figure 8B) (p < 0.05). In the fasted state, Liver-ChREBP KO mice had increased expression of the glucagon receptor (2.1-fold) (Figure 8A) (p < 0.01). Similar to in WAT, Liver-ChREBP KO mice had increased expression of PEPCK in BAT in the fed state (2.6-fold) (Figure 8B) (p < 0.001). Interestingly, FOXO1 was upregulated in Liver-ChREBP KO mice in both fed (2.3-fold) and fasted (1.8-fold) states (Figure 8A,B) (p < 0.05). FOXO1 is a negative regulator of ChREBP and has also been shown to regulate energy expenditure in brown adipose tissue. Interestingly, the changes in PEPCK, FGF21 and FOXO1 observed in the fed state were no longer detectable after high-fat diet feeding (Supplemental Figure 14B).

## 4 Discussion

In this study, we characterized a novel mouse model of hepatic ChREBP deletion to investigate the importance of this factor in glucose homeostasis and insulin sensitivity. We found liver ChREBP is essential for maintaining whole-body glucose homeostasis and hepatic insulin sensitivity. Liver-ChREBP KO mice had reduced fat mass but gained similar weight when fed a high-fat diet. Regardless of diet, these mice displayed glucose intolerance and insulin resistance with specific impairments in the suppression of hepatic glucose production. Along with the reduced liver insulin sensitivity, hepatic ChREBP deletion impaired the lipogenic transcriptional response to fasting and feeding suggesting a lack of appropriate compensation by alternate lipogenic transcription factors to glucose sensing. Apart from these effects on the liver, there were also dramatic gene expression changes evident in both white and brown adipose tissues. Finally, although ChREBP deletion did protect against carbohydrate-diet induced hepatic steatosis this was not protective in terms of metabolic health. The alterations in liver in response to ChREBP deletion have wide-ranging effects on whole body glucose homeostasis and can induce specific changes in WAT and BAT to affect lipid homeostasis.

Perhaps the most striking effect of hepatic ChREBP deletion was the impairment of hepatic insulin sensitivity. Liver-ChREBP KO mice displayed glucose intolerance, which was most likely due to insulin no longer being able to suppress hepatic glucose production (HGP) effectively. Importantly, this impairment was also seen after high-fat diet feeding, despite no differences in body composition. This suggests that ChREBP action in the liver is important in maintaining hepatic insulin sensitivity. This contradicts human association studies showing an association of insulin resistance with increased liver ChREBP [7], [10] and suggests that the increase seen in obesity is most likely compensatory. Furthermore, it contradicts suggestions that ChREBP deletion is beneficial in states of lipid overload as has previously been suggested [18], [20]. Hyperglycemia and poor blood glucose control contribute to various microvascular and macrovascular complications in diabetes and so are important processes to understand. How ChREBP influences insulin sensitivity is unclear, but one potential mechanism could be production of insulin-sensitizing lipid mediators. Recent studies have shown ChREBP synthesizes a novel class of insulin-sensitizing and anti-inflammatory lipids, FAHFAs, in adipose tissue [16]. These FAHFAs were shown to increase insulin-stimulated glucose transport in adipocytes via activation of the G-protein coupled receptor GPR120 [16]. Whether liver-derived FAHFAs could influence hepatic insulin sensitivity in a similar way remains to be tested. However, activation of GPR120 using an agonist does improve insulin sensitivity in liver [21], which suggests hepatic ChREBP synthesized FAHFAs could act in a similar way. It must be noted that there are numerous other transcription factors that can induce transcription of glycolytic and lipogenic genes, including upstream stimulatory factors (USFs), sterol regulatory element-binding protein 1c (SREBP1c), liver X receptors (LXRs), hepatocyte nuclear factor 4 (HNF4α), and c-myc [22], [23], [24]. For instance, both c-myc and HNF4α can bind to the L-PK promoter [22], [23]. However, although these factors are recruited to the L-PK promoter, they are not transactivated by glucose. Therefore, although alternate transcription factors likely compensate for the deletion of ChREBP, as suggested by the mRNA expression of the supposed ChREBP-specific target gene L-PK, it is likely that these transcription factors cannot respond appropriately to glucose. This is supported by ChREBP target gene expression being induced in Liver-ChREBP KO mice inappropriately in the fasting state. Furthermore, previous studies have shown ChREBP deletion completely reverses the elevation in FAHFAs seen in mice with adipose tissue over-expression of GLUT4 [16]. Additionally, high-fat diet Liver-ChREBP KO mice have reduced target gene expression, suggesting compensation can no longer occur with dietary overload. This includes reductions in lipogenic genes as well as FGF21, which is a known ChREBP target gene that can influence insulin sensitivity [25], [26]. This suggests that Liver-ChREBP KO mice cannot respond appropriately to feeding, thus impairing hepatic lipid homeostasis, potentially impairing hepatic insulin action via lack of a favorable autocrine signal, resulting in elevated HGP and impaired glucose homeostasis.

Deletion of hepatic ChREBP may also affect indirect mechanisms of HGP regulation. It is known that insulin can directly suppress HGP through hepatic Akt signaling resulting in inhibition of FOXO1. Yet, studies have shown insulin acts through direct and indirect pathways in humans to suppress HGP [27]. Various mouse models have shown that if the insulin receptor or Akt is deleted, along with FOXO1, insulin is still able to regulate HGP through indirect mechanisms [28], [29]. This can be centrally mediated by hypothalamic neurons that can respond to glucose to regulate HGP [30], [31]. Furthermore, insulin's ability to suppress HGP has been shown to be at least partly dependent on its inhibition of lipolysis in WAT [32]. In fact, recently Perry et al. have shown that the main mechanism by which insulin suppresses gluconeogenesis is by suppressing lipolysis of WAT. This reduces fatty acid flux thereby reducing hepatic acetyl-coA and decreasing pyruvate carboxylase activity, resulting in reduced conversion of pyruvate to glucose [33]. The authors show that diet induced obesity impairs this pathway due to increased inflammatory mediators in adipose and serum [33]. Interestingly, Liver-ChREBP KO mice have increased serum inflammatory markers PAI-1 and resistin, suggestive of a heightened inflammatory state. This may be due to a reduction in anti-inflammatory mediators from liver, such as FAHFAs or another metabolite, acting in an endocrine manner to increase inflammation and interfere with insulin action on adipose tissue. However, it must be noted that we did not see an increase in the mRNA expression of inflammatory markers in adipose tissue. Perhaps, in Liver-ChREBP KO mice, the reduction in hepatic insulin-sensitizing lipids can result in a reduced efficacy of insulin to suppress lipolysis in WAT. On a chow diet, Liver-ChREBP KO mice have a reduced fat mass and smaller adipocyte size. Furthermore, on a high-fat diet Liver-ChREBP KO mice have elevated serum non-esterified fatty acid levels (Supplemental Figure 15). This is suggestive of increased lipolysis in these mice. Liver-ChREBP KO mice show a striking elevation in PEPCK mRNA in WAT. In WAT, PEPCK is important in glyceroneogenesis and therefore re-esterification of fatty acids. It has previously been shown that PEPCK is controlled at the level of transcription [34], thus the increased expression seen is a reliable measure of increased activity. Importantly, re-esterification of fatty acids increases with increased lipolysis as free fatty acids are recycled back to triglycerides. It has been shown that the fraction of free fatty acids released during lipolysis that are recycled remains relatively constant and it is the rate of triglyceride/fatty acid cycling that changes during fasting and fed states [35]. Therefore, the dramatic increase in PEPCK seen in Liver-ChREBP KO mice in both fed and fasted states is likely due to increased fatty acid cycling due to increased lipolysis. Whether this is causing the impairment in hepatic insulin sensitivity, or is a consequence of it, remains to be determined. Regardless of the specific mediator responsible it is clear that specific deletion of hepatic ChREBP can induce metabolic changes not only in the liver but also in white adipose tissue.

Hepatic ChREBP deletion resulted in profound alterations in brown adipose tissue gene expression. Strikingly, the induction of BAT ChREBPβ expression in the fed state was almost completely abolished in Liver-ChREBP KO mice. This was accompanied by a trend towards decreased lipogenic gene expression in both fed and fasted states. The role of ChREBP in BAT is not completely understood, but it has been shown to induce lipogenesis and is involved in a feedback loop with PPARα to coordinate appropriate lipogenesis and lipolysis [36]. This may be important in thermogenesis as *de novo* lipogenesis in brown adipose tissue increases supply of fatty acid substrates. In fact, although acute activation of β3-adrenoceptors, which mediate sympathetic activation of thermogenesis, suppresses *de novo* lipogenic genes chronic administration increases them [37]. In the short term β3-adrenoceptor activation increases lipolysis and promotes fatty acid oxidation and uncoupling but chronic stimulation will simultaneously increase lipid synthesis allowing continual substrate supply [37]. Therefore, the impairment in BAT ChREBP may impair the coordination of lipolysis and lipogenesis resulting in decreased substrate availability and therefore decreased uncoupling and thermogenesis. What is causing the reduction in ChREBP is unclear but may be due to impaired insulin action in BAT. It is plausible that reductions in favorable metabolites from liver would also influence BAT. In support of this, there is increased expression of FOXO1 in both the fed and fasted states in BAT. Insulin is known to inhibit FOXO1 so the elevation seen may be due to reduced efficacy of insulin. Furthermore, FOXO1 can inhibit ChREBP activity and protein stability in hepatocytes and pancreatic β cells [38], [39]. Whether FOXO1 also plays a role in regulation of BAT ChREBP remains to be seen. Insulin also inhibits the transcriptional repressor Oct-1, which has been shown to inhibit ChREBP mRNA and protein expression in a liver cell line [40]. Therefore, the reduction seen in ChREBP could be due to decreased insulin action at BAT resulting in elevated FOXO1 and Oct-1. Liver-ChREBP KO mice also had decreased expression of FGF21 in BAT. Interestingly, activation of thermogenesis induces FGF21 mRNA expression in brown adipose tissue followed by release from brown adipocytes [41], [42]. This again suggests that thermogenesis may be decreased in Liver-ChREBP KO mice and further highlights a potential role for ChREBP in regulating BAT FGF21. It will be important to investigate whether thermogenesis is altered in Liver-ChREBP KO mice and future investigations into the role ChREBP in BAT thermogenesis would be beneficial.

Although hepatic ChREBP deletion can protect mice from carbohydrate-diet induced hepatic steatosis this is not protective in terms of metabolic health. Despite reductions in liver triglyceride, Liver-ChREBP KO mice fed a high-carbohydrate diet display worsened glucose tolerance and increased serum PAI-1 and resistin (Supplemental Figures 9 and 15). This supports the view that hepatic steatosis is not necessarily detrimental, and, in fact, increasing hepatic *de novo* lipogenesis may be beneficial [17], [43]. Adenoviral-mediated over-expression of ChREBP in liver of mice improves insulin sensitivity when fed a high-fat diet despite worsened hepatic steatosis [17]. Whether this is due to increased favorable lipids or decreased harmful lipids is unclear and may depend on the nutritional context. Although control and Liver-ChREBP KO mice both display similar liver triglyceride deposition on chow and high-fat diets the KO mice have impaired glucose tolerance and hepatic insulin resistance. A lipidomic analysis of Liver-ChREBP KO mice on chow, high-carbohydrate, and high-fat diets would be insightful.

By examining the effects of hepatic ChREBP deletion, a complex inter-tissue communication network to maintain metabolic homeostasis can be observed. Deletion of ChREBP in liver impairs the normal response to fasting and feeding as observed by the lipogenic gene expression profiles. Furthermore, Liver-ChREBP KO mice have reduced hepatic fatty acid oxidation, suggesting alterations in substrate utilization. This is seen at a whole body level with changes in the respiratory exchange ratio reflecting increased oxidation of carbohydrates, particularly after fasting. The liver is the central organ of glucose and lipid metabolism, so it is unsurprising that alterations here would impact other metabolic tissues. Although adipose tissue and adipokines have recently garnered the most attention in their ability to influence whole body insulin and glucose homeostasis, the liver undoubtedly plays a role as well. In fact, this has been shown previously where lipids from the liver can regulate *de novo* lipogenesis and fatty acid oxidation in WAT [44]. Furthermore, FGF21 is released by the liver in the fasting state to regulate the appropriate response in other tissues including WAT, where it stimulates lipolysis [45], and BAT, where it increases glucose uptake [26]. Cycling between FGF21 and hepatic ChREBP has been shown to be important in coordinating the appropriate central and peripheral responses to carbohydrate and fructose feeding in mice [46], [47]. Interestingly, Liver-ChREBP KO mice have decreased FGF21 in the fasted state but increased FGF21 in the fed state again reflecting the impairment in an appropriate glucose response in these mice. These impairments influence both white and brown adipose tissues. Liver-ChREBP KO mice have reduced WAT mass as well as inappropriate upregulation of lipogenic genes in the fasted state and increased PEPCK indicative of increased fatty acid cycling. Whereas in BAT, Liver-ChREBP KO mice show reduced ChREBP and FGF21 expression and increased FOXO1 expression. Furthermore, after high-fat diet feeding, Liver-ChREBP KO mice have a reduction in hepatic FGF21 mRNA expression. These alterations in liver FGF21 may play a role in the worsened metabolic homeostasis seen with high-fat diet feeding in these mice. The liver is vital in conveying information about the metabolic state of the body to other tissues in order to coordinate tissue responses. Impairments in the appropriate glucose-sensing response due to hepatic ChREBP deletion lead to impairments in lipid and glucose homeostasis. This may be due to impaired synthesis of FAHFAs but may also be due to altered expression of various other metabolites. A comprehensive metabolomic screening of Liver-ChREBP KO mice could help identify novel mediators and is a focus for future studies.

In conclusion, we have characterized a novel mouse model to examine the importance of hepatic ChREBP on whole body glucose homeostasis and insulin sensitivity. Liver ChREBP is vital in maintaining hepatic insulin sensitivity and coordinating the appropriate responses to fasting and feeding through glucose sensing. Future investigations to identify the mediators of these responses may reveal novel pathways that could be exploited for therapeutic development for hyperglycemia and diabetes.

## Appendix A Supplementary data

mmc1 mmc1

mmc2 mmc2
