# Supplementary material for: Deletion of hepatic carbohydrate response element binding protein (ChREBP) impairs glucose homeostasis and hepatic insulin sensitivity in mice
Source: Mol Metab. 2017 Jul 18;6(11):1381–94. doi: 10.1016/j.molmet.2017.07.006 (PMC5681238; doi:10.1016/j.molmet.2017.07.006)
Supplement: mmc1 [file mmc1.docx]

**Supplemental Table 1. Detailed comparison of high-fat (HFD) and high-carbohydrate (HCD) diets**

| **Ingredient** | **HFD SF04-001** | **HCD SF13-067** |
| --- | --- | --- |
| Casein (Acid) | 233 g/Kg | 185 g/Kg |
| Sucrose | 201 g/Kg | 201 g/Kg |
| Lard | 207 g/Kg | 20.1 g/Kg |
| High Linoleic Safflower Oil |  | 10.4 g/Kg |
| Soya Bean Oil | 29 g/Kg | 4.3 g/Kg |
| Cellulose | 58 g/Kg | 58 g/Kg |
| Wheat Starch | 92 g/Kg | 354 g/Kg |
| Dextrinised Starch | 117 g/Kg | 117 g/Kg |
| DL Methionine | 3.5 g/Kg | 2.8 g/Kg |
| Calcium Carbonate | 6.4 g/Kg | 5.1 g/Kg |
| Sodium Chloride | 2.6 g/Kg | 2.1 g/Kg |
| AIN93 Trace Minerals | 1.6 g/Kg | 1.3 g/Kg |
| Potassium Citrate | 19.2 g/Kg | 15.3 g/Kg |
| Dicalcium Phosphate | 15.1 g/Kg | 12 g/Kg |
| Potassium Sulphate | 1.6 g/Kg | 1.3 g/Kg |
| Choline Chloride (75%) | 1.3 g/Kg | 1.0 g/Kg |
| AIN93 Vitamins | 12 g/Kg | 9.3 g/Kg |

**Statistical Analysis of CLAMS data**

As outlined in the methods section, Liver-ChREBP KO (KO) and wild type (WT) mice were individually housed in metabolic chambers (CLAMS, Columbus Instruments) in order to assess metabolic activity. They were acclimated for 48 hours before data was collected at time 0 in Supplementary Graphs (1-5 below). Raw data is plotted for each mouse (thin coloured lines) and the mean of the group (thick lines; yellow KO and green, WT) were plotted for the day (left side) and night (right side) period. Oxygen consumption, carbon dioxide production and ambulatory activity were measured, and RER and energy expenditure were calculated. A statistical analysis was performed on this data using population average modeling (Wang 2014) adjusting for bodyweight, lean mass, fat mass over the dark/light period and results are presented in a table for each variable.

**Reference**

M Wang, “ Generalized Estimating Equations in Longitudinal Data Analysis: A Review and recent developments” Advances in Statistics Vol 2014 Article ID 303728, 11 pages
<http://dx.doi.org/10.1155/2014/303728>

|  | Average difference between mouse type | 95% CI | P Value |
| --- | --- | --- | --- |
| VO2 adjusted day/night | -412.2 | -665.4 to -159.0 | 0.001 |
| VO2 adjusted day/night, body weight | 4.7 | -370.5 to 380.0 | 0.98 |
| VO2 adjusted day/night, lean mass | -404.0 | -763.0 to -45.3 | 0.03 |
| VO2 adjusted day/night, fat mass | -211.1 | -467.2 to 45.1 | 0.11 |

**Supplementary Fig 1 Oxygen consumption of Liver-ChREBP KO and WT mice.** Raw data is plotted for each individual mouse (thin coloured lines) and for the mean of the group (thick lines; yellow L-ChREBP KO and green, WT) were plotted for the day (left side) and night (right side) period.

|  | Difference between mouse type | 95% CI | P Value |
| --- | --- | --- | --- |
| VCO2 adjusted day/night | -461.0 | -749.5 to -172.4 | 0.002 |
| VCO2 adjusted day/night, body weight | -77.5 | -544.9 to 390.0 | 0.75 |
| VCO2 adjusted day/night, lean mass | -494.1 | -902.1 to -86.2 | 0.02 |
| VCO2 adjusted day/night, fat mass | -256.4 | -564.0 to 51.1 | 0.10 |

**Supplementary Fig 2 Carbon Dioxide production of Liver-ChREBP KO and WT mice.** Raw data is plotted for each individual mouse (thin coloured lines) and for the mean of the group (thick lines; yellow L-ChREBP KO and green, WT) were plotted for the day (left side) and night (right side) period.

|  | Difference between mouse type | 95% CI | P Value |
| --- | --- | --- | --- |
| RER adjusted day/night | -0.015 | -0.041 to 0.012 | 0.28 |
| RER adjusted day/night, body weight | -0.015 | -0.065 to 0.034 | 0.54 |
| RER adjusted day/night, lean mass | -0.025 | -0.062 to 0.012 | 0.19 |
| RER adjusted day/night, fat mass | -0.011 | -0.044 to 0.023 | 0.53 |

**Supplementary Fig 3 Respiratory Quotient (RER) of Liver-ChREBP KO and WT mice.** Derived data is plotted for each individual mouse (thin coloured lines) and for the mean of the group (thick lines; yellow L-ChREBP KO and green, WT) were plotted for the day (left side) and night (right side) period.

|  | Difference between mouse type | 95% CI | P Value |
| --- | --- | --- | --- |
| Heat adjusted day/night | -2.12 | -3.55 to -0.69 | 0.004 |
| Heat adjusted day/night, body weight | 0.23 | -1.89 to 2.36 | 0.83 |
| Heat adjusted day/night, lean mass | -2.11 | -4.14 to -0.08 | 0.04 |
| Heat adjusted day/night, fat mass | -0.97 | -2.40 to 0.47 | 0.19 |

**Supplementary Fig 4 Heat generated by Liver-ChREBP KO and WT mice.** Derived data is plotted for each individual mouse (thin coloured lines) and for the mean of the group (thick lines; yellow L-ChREBP KO and green, WT) were plotted for the day (left side) and night (right side) period.

|  | Difference between mouse type | 95% CI | P Value |
| --- | --- | --- | --- |
| Activity adjusted day/night | 25.27 | -45.77 to 96.30 | 0.49 |
| Activity adjusted day/night, body weight | 41.11 | -89.87 to 172.09 | 0.54 |
| Activity adjusted day/night, lean mass | -9.08 | -105.9 to 87.73 | 0.85 |
| Activity adjusted day/night, fat mass | 54.50 | -360.48 to -84.03 | 0.002 |

**Supplementary Fig 5 Activity of Liver-ChREBP KO and WT mice.** Raw data is plotted for each individual mouse (thin coloured lines) and for the mean of the group (thick lines; yellow L-ChREBP KO and green, WT) were plotted for the day (left side) and night (right side) period.


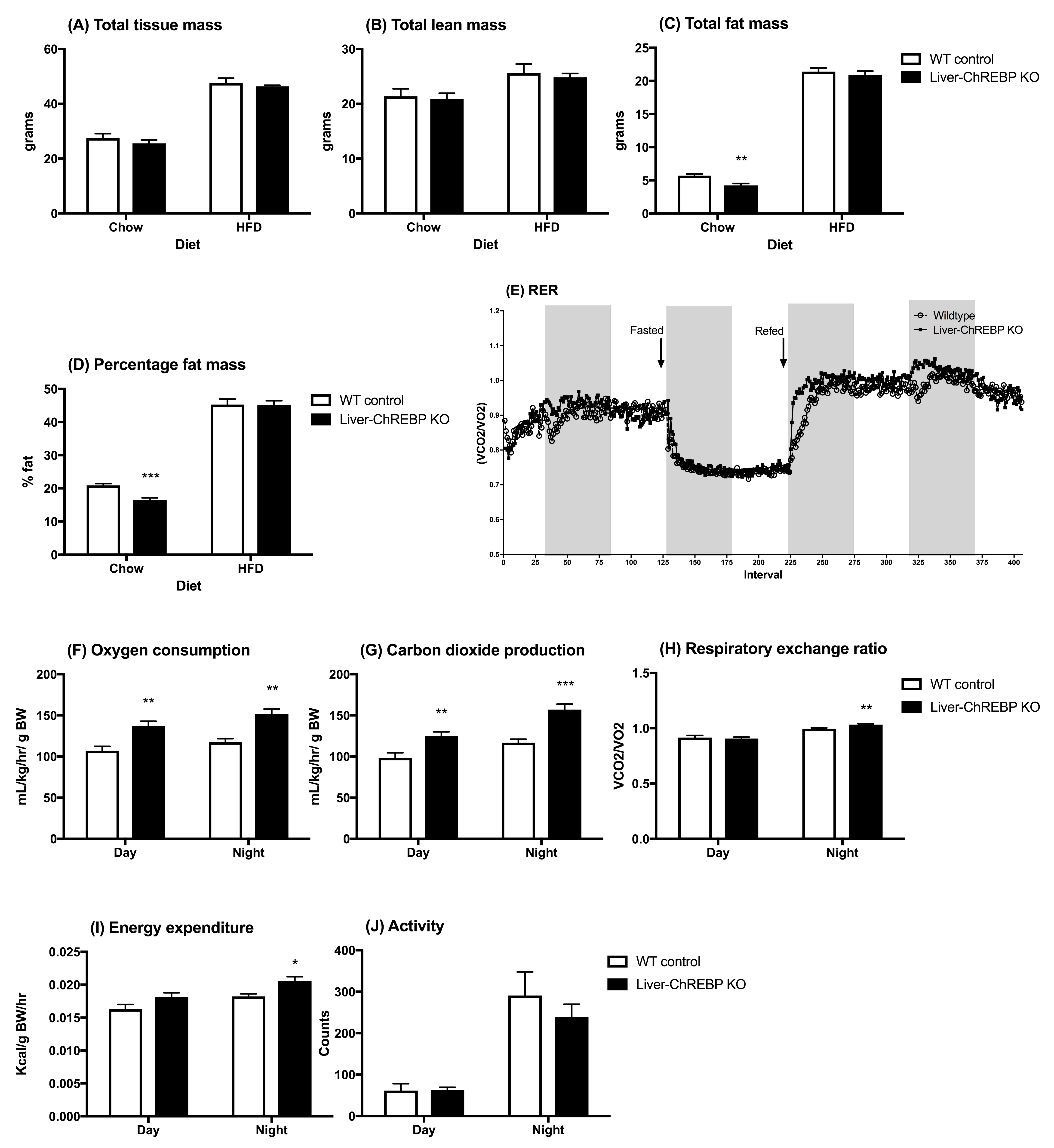


**Supplemental Figure 6. Body composition and metabolic activity of Liver-ChREBP KO mice**A. Total tissue mass, B. Total lean mass, C. Total fat mass, and D. Percentage fat mass in Liver-ChREBP KO mice and WT or Fl/Fl control mice fed a chow or HFD (n=4-7 per group). E. Data from CLAMS metabolic cages shows the calculated respiratory quotient (RER) over a 5-day fasting-refeeding paradigm in chow-fed Liver-ChREBP KO and WT mice (n=6 per group). Results for A-D are expressed as mean ± SEM and a statistical analysis by unpaired t-test within each dietary condition (,**: p<0.01, ***: p<0.001).


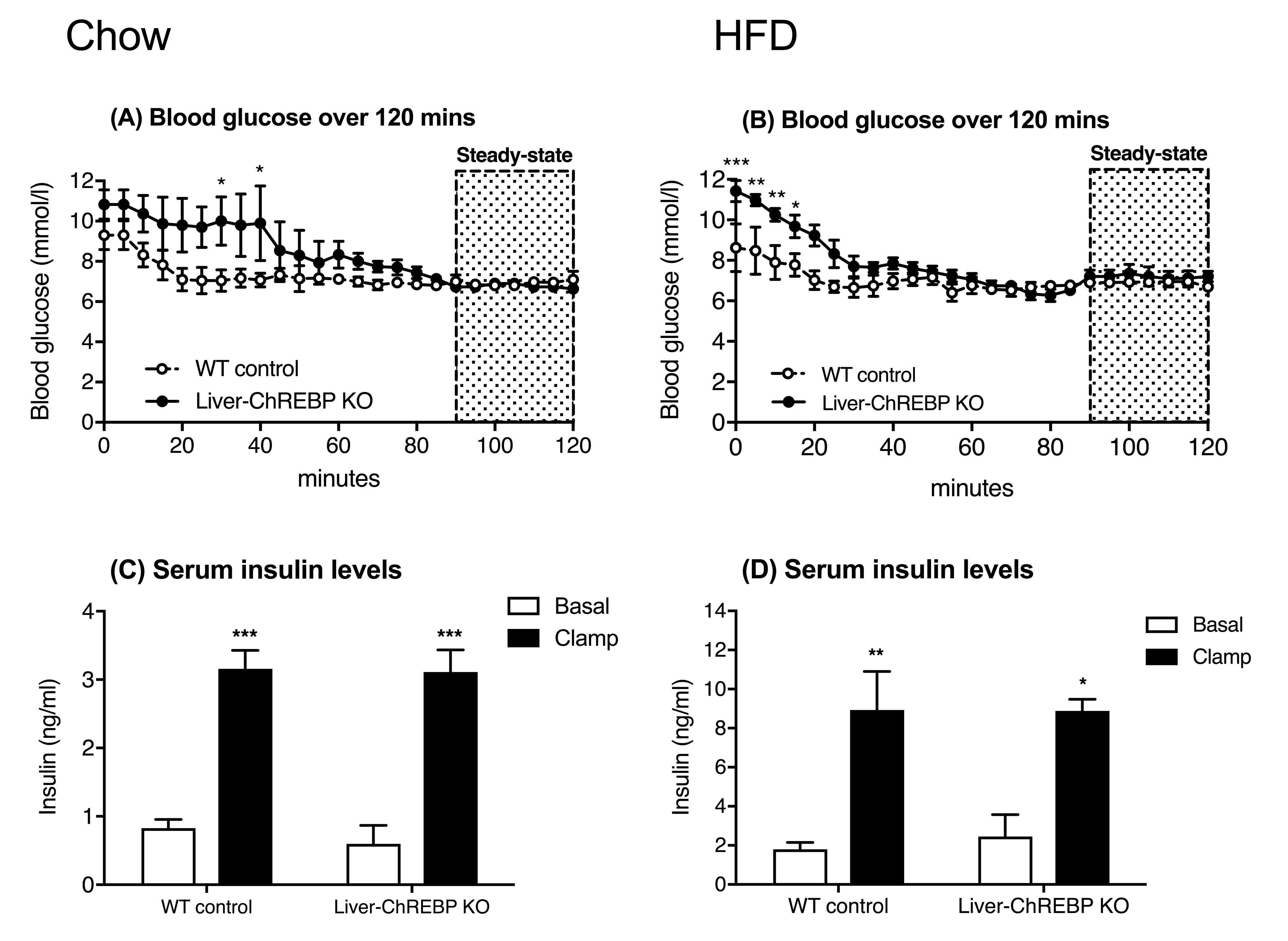


**Supplemental Figure 7. Blood glucose and insulin levels during hyperinsulinemic-euglycemic clamps in Liver-ChREBP KO mice**Blood glucose levels over 120 minutes during hyperinsulinemic-euglycemic clamp in Liver-ChREBP KO and WT control mice (n=4 per group) on chow (A) or high-fat diet (B).
Serum insulin levels prior and during hyperinsulinemic-euglycemic clamp in Liver-ChREBP KO and WT control mice (n=4 per group) on chow (C) or high-fat diet (D).
Results expressed as mean ± SEM. Statistical analysis by two-way ANOVA followed by Tukey’s post-hoc test (*: p<0.05, **: p<0.01, ****: p<0.0001).


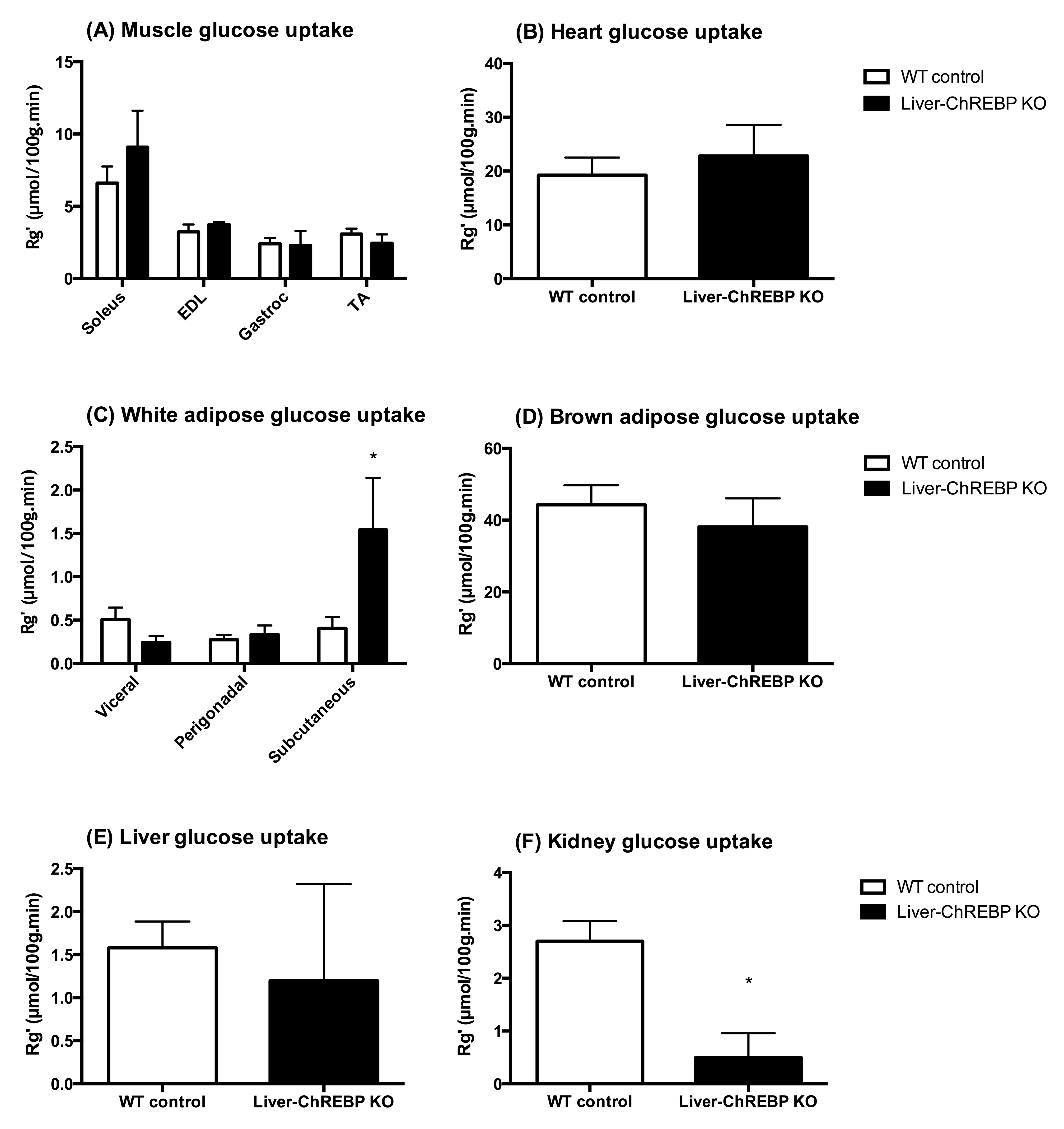


**Supplemental Figure 8. 2-deoxyglucose uptake in Liver-ChREBP KO mice**2-deoxyglucose uptake in various tissues in Liver-ChREBP KO and WT control mice (n=4 per group). Results expressed as mean ± SEM. Statistical analysis by two-way ANOVA followed by Tukey’s post-hoc test (C), or unpaired t-test (F) (*: p<0.05).


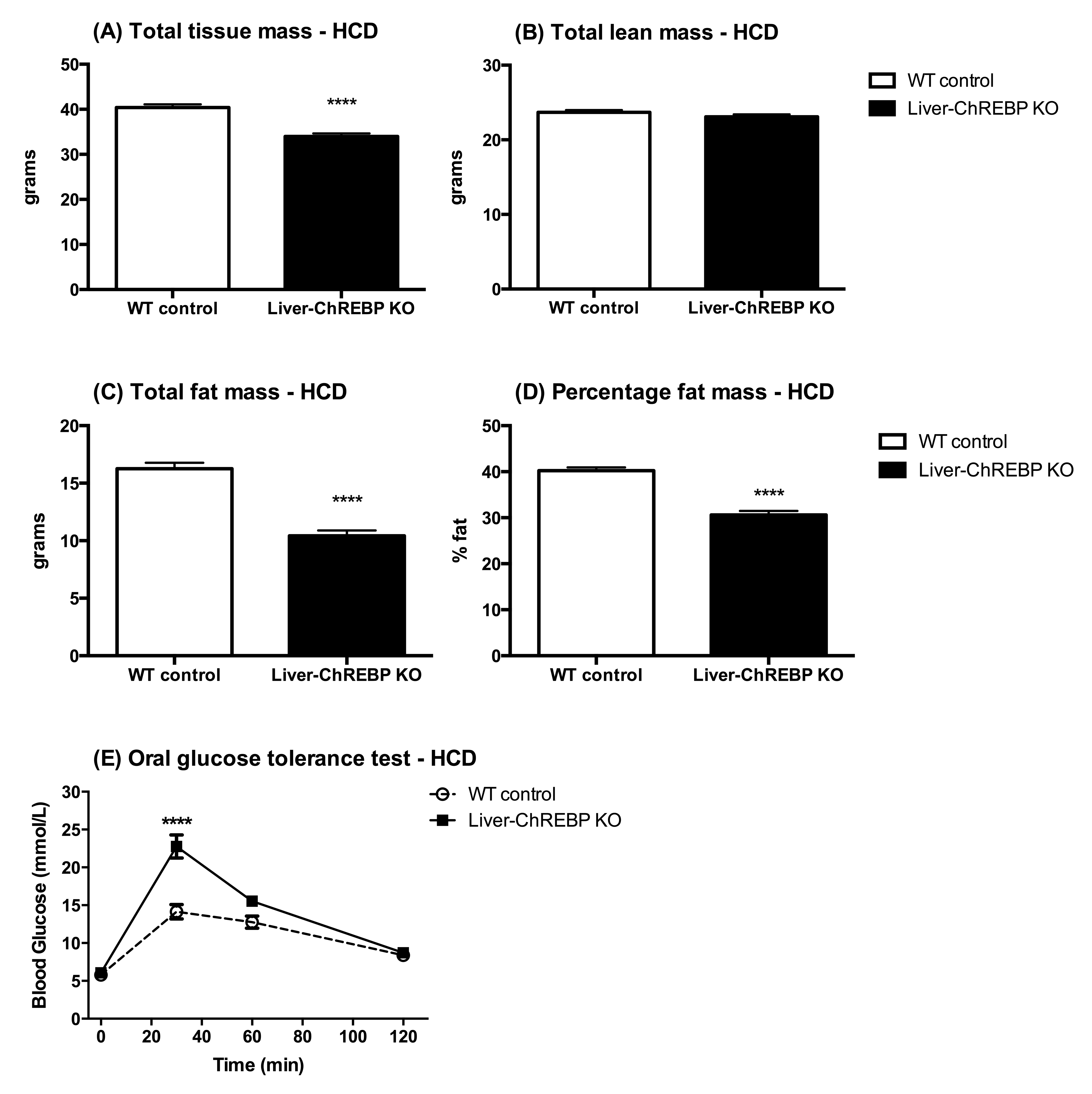


**Supplemental Figure 9. Body composition of Liver-ChREBP KO mice in response to high-carbohydrate diet**
Effect of HCD on **A.** Total tissue mass, **B.** Total lean mass, **C.** Total fat mass, and **D.** Percentage fat mass of Liver-ChREBP KO and WT mice after 8 weeks on diet (n=7 per group). **E.** Oral glucose tolerance test (oGTT) in Liver-ChREBP KO and WT mice after 8 weeks on HCD (n=7 per group).
Results expressed as mean ± SEM. Statistical analysis by unpaired t-test (A, C, D) or by two-way ANOVA followed by Tukey’s post-hoc test (E) (****: p<0.0001).


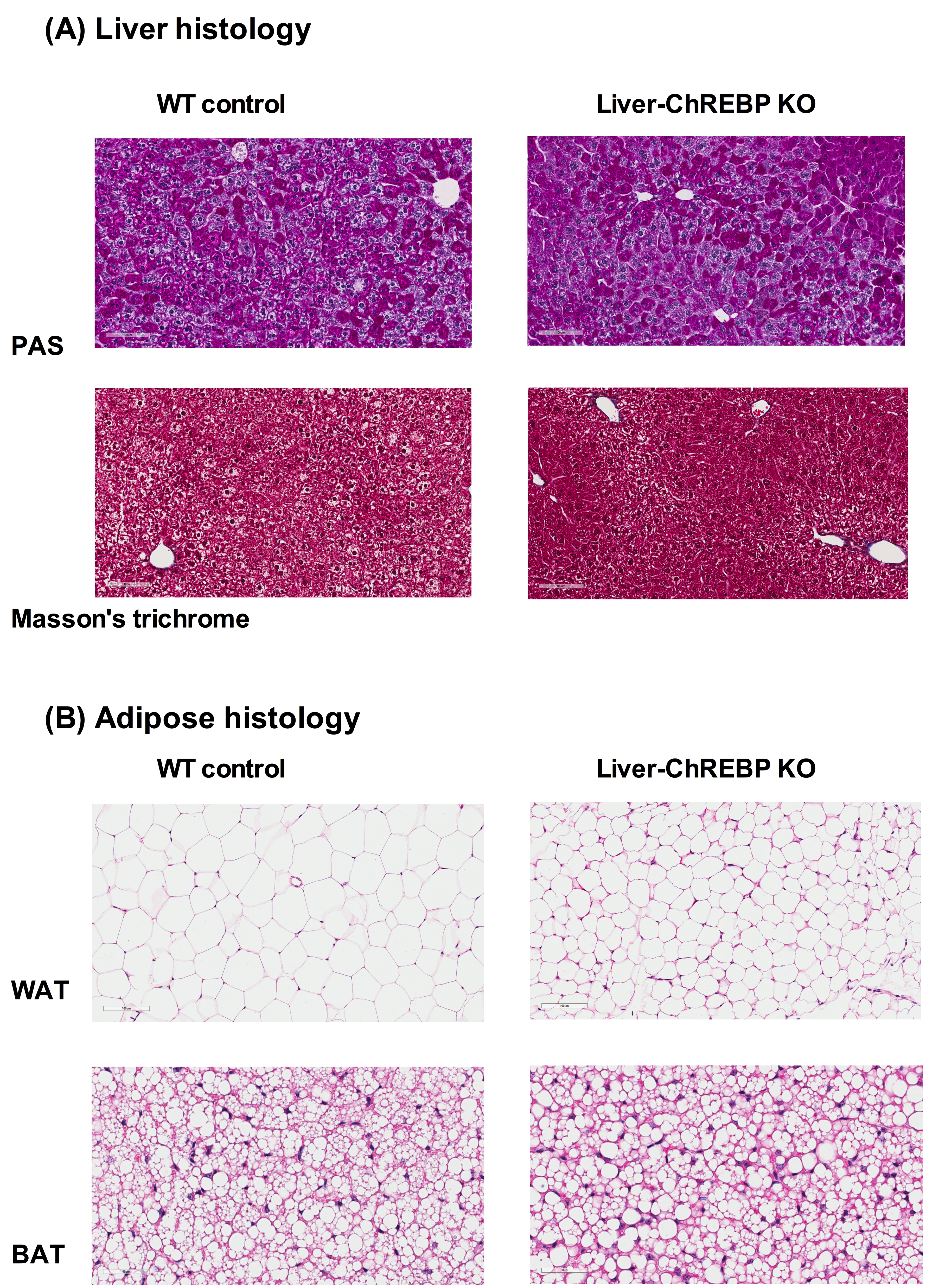


**Supplemental Figure 10. Liver and adipose histology in Liver-ChREBP KO mice**(A) Liver histology showing representative PAS and Masson’s trichrome stained liver sections from Liver-ChREBP KO and WT control mice. (B) White and brown adipose tissue histology showing representative H&E stained adipose sections from Liver-ChREBP KO and WT control mice. Magnification = 20x.


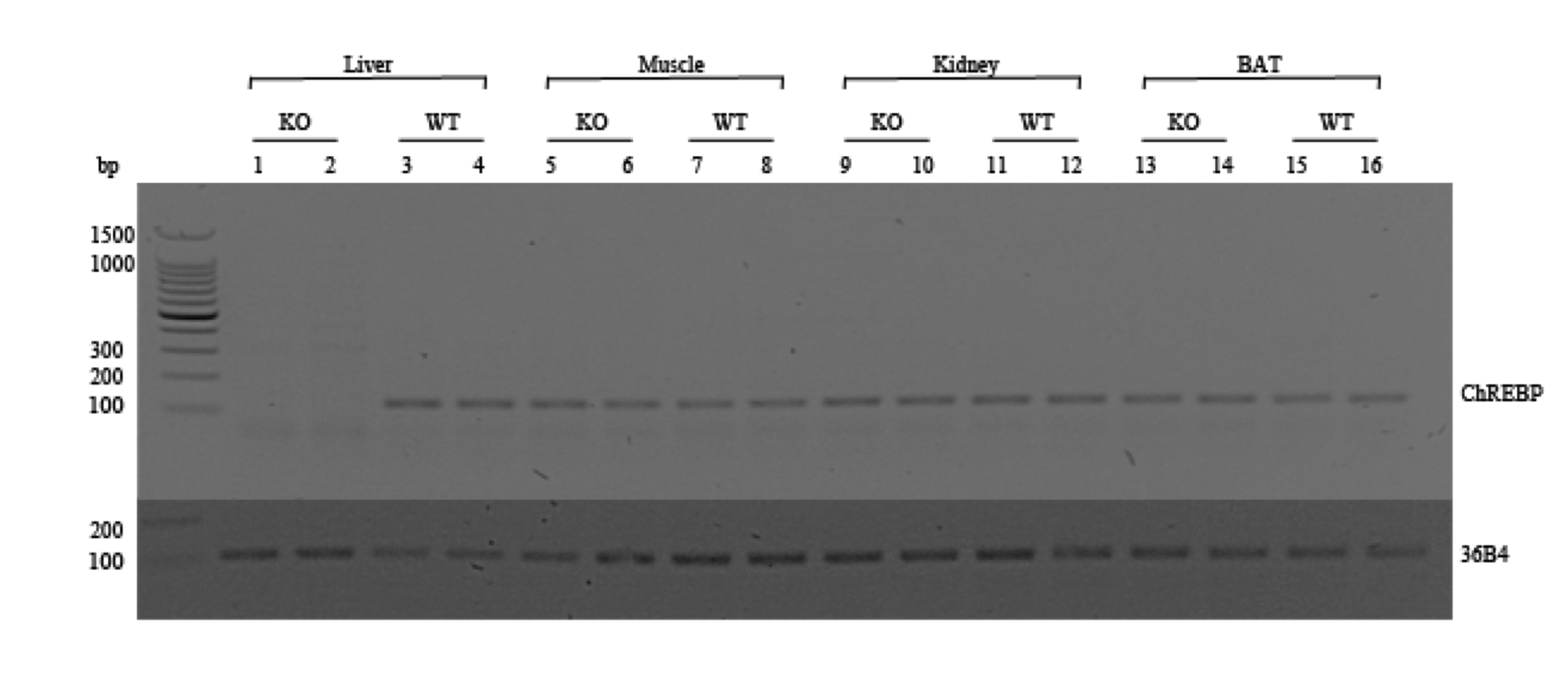


**Supplemental Figure 11. ChREBP expression in Liver-ChREBP KO mice**PCR products of ChREBP and 36B4 in liver, muscle, kidney and BAT in Liver-ChREBP KO (KO) and WT control (WT) mice.


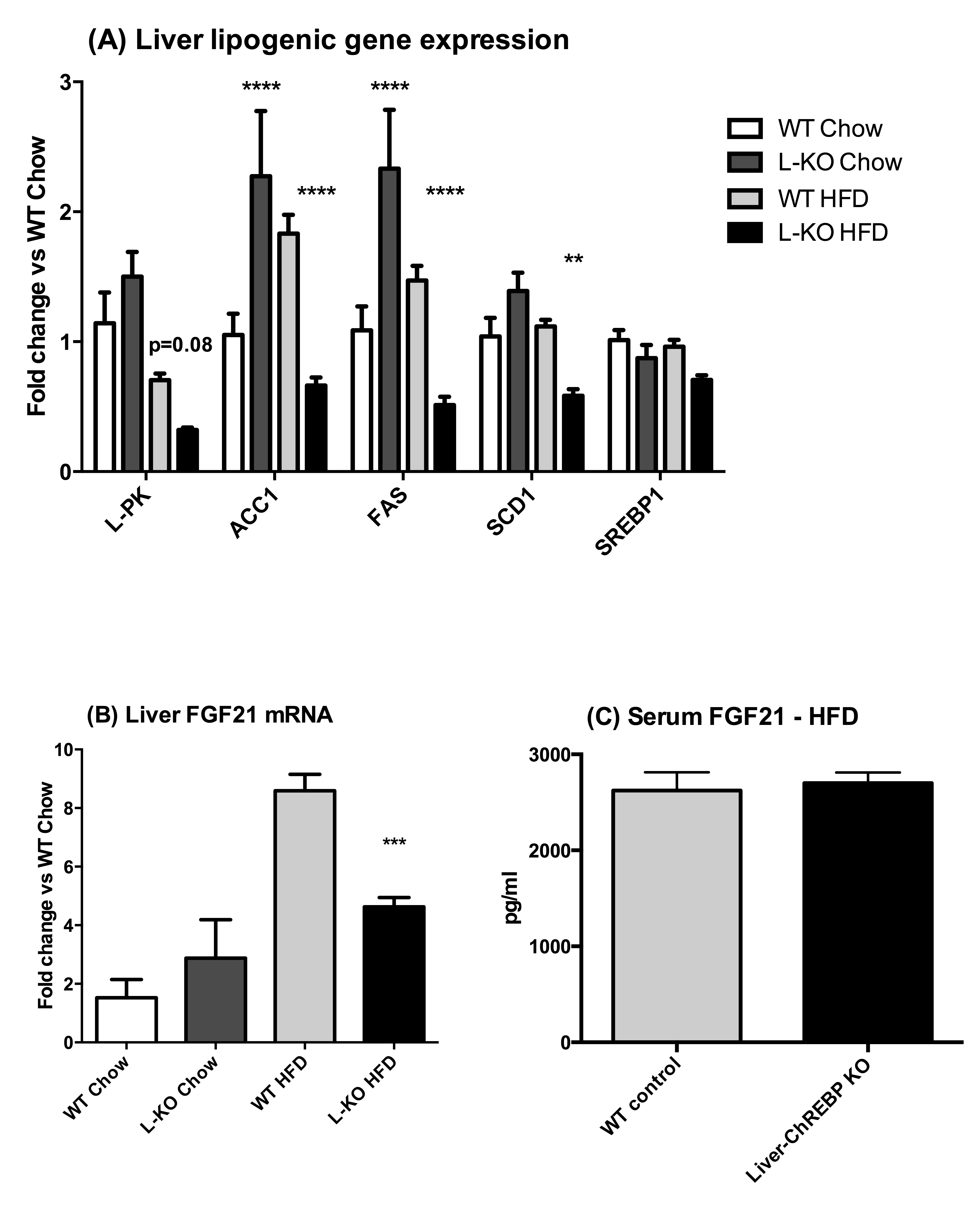


**Supplemental Figure 12. Gene expression in liver and serum FGF21 of chow or high-fat diet fed Liver-ChREBP KO mice**
(A) Lipogenic gene expression in liver of Liver-ChREBP KO and WT control mice fed either a chow or high-fat diet. (B) FGF21 mRNA expression in liver of Liver-ChREBP KO and WT control mice fed either a chow or high-fat diet. Results were normalized to expression of one of 3 housekeeping genes; 36B4, Hprt or Pgk1; and then shown as fold change versus the WT chow group. (C) Serum FGF21 in high-fat diet fed Liver-ChREBP KO and WT control mice.
Results are expressed as mean ± SEM. Statistical analysis was by two-way ANOVA followed by Tukey’s post-hoc test. (**: p<0.01, ***: p<0.001, ****: p<0.0001 denoting significance between genotypes and within a dietary treatment).


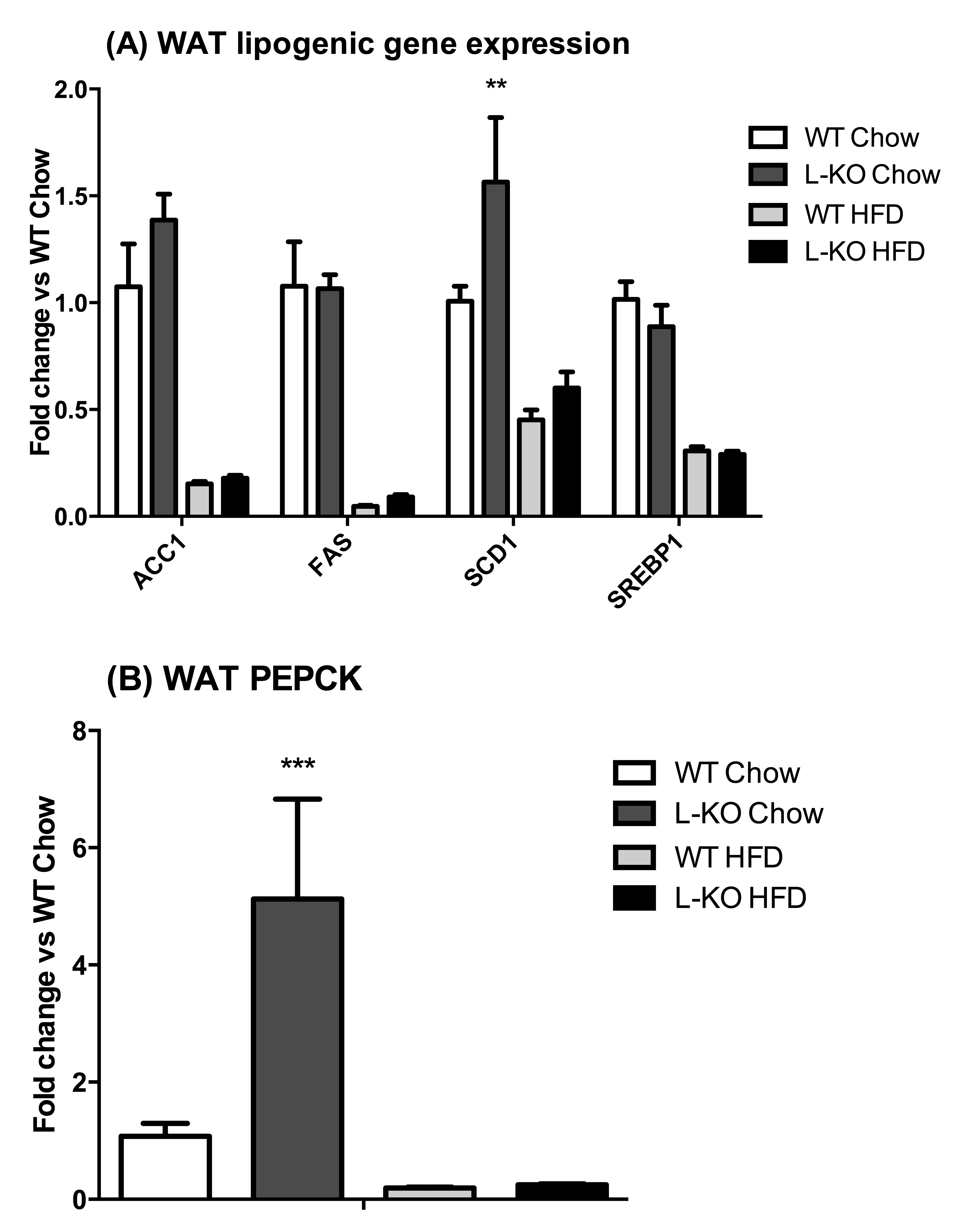


**Supplemental Figure 13. Gene expression in white adipose tissue of chow and high-fat diet fed Liver-ChREBP KO mice**
(A) Lipogenic gene expression in WAT of Liver-ChREBP KO and WT control mice fed either a chow or high-fat diet. (B) PEPCK mRNA expression in WAT of Liver-ChREBP KO and WT control mice fed either a chow or high-fat diet. Results were normalized to expression of one of 3 housekeeping genes; 36B4, Hprt or Pgk1; and then shown as fold change versus the WT chow group.
Results are expressed as mean ± SEM. Statistical analysis was by two-way ANOVA followed by Tukey’s post-hoc test. (**: p<0.01, ***: p<0.001 denoting significance between genotypes and within a dietary treatment).


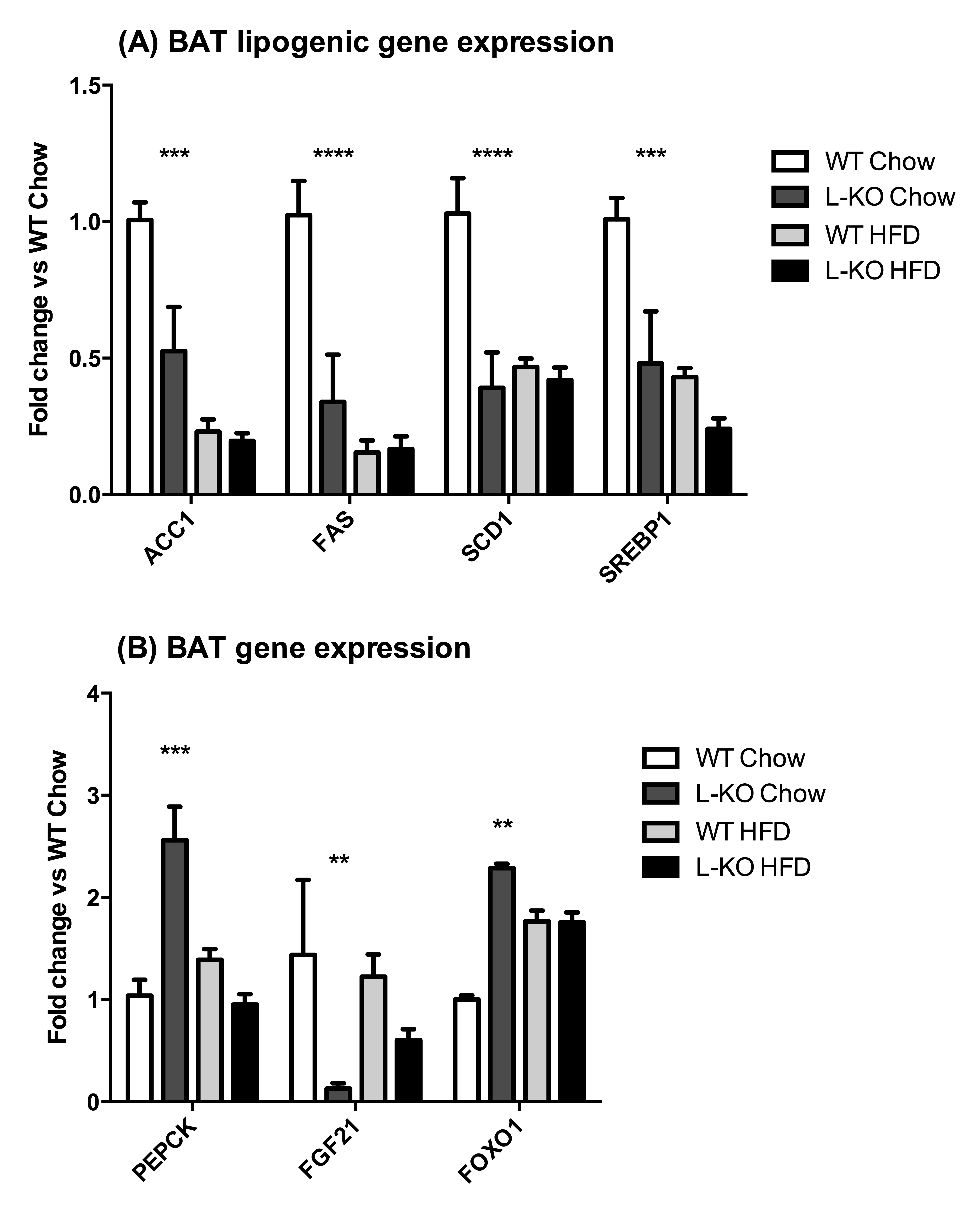


**Supplemental Figure 14. Gene expression in brown adipose tissue of chow and high-fat diet fed Liver-ChREBP KO mice**
(A) Lipogenic gene expression in BAT of Liver-ChREBP KO and WT control mice fed either a chow or high-fat diet. (B) PEPCK. FGF21 and FOXO1 mRNA expression in BAT of Liver-ChREBP KO and WT control mice fed either a chow or high-fat diet. Results were normalized to expression of one of 3 housekeeping genes; 36B4, Hprt or Pgk1; and then shown as fold change versus the WT chow group.
Results are expressed as mean ± SEM. Statistical analysis was by two-way ANOVA followed by Tukey’s post-hoc test. (**: p<0.01, ***: p<0.001, ****: p<0.0001 denoting significance between genotypes and within a dietary treatment).


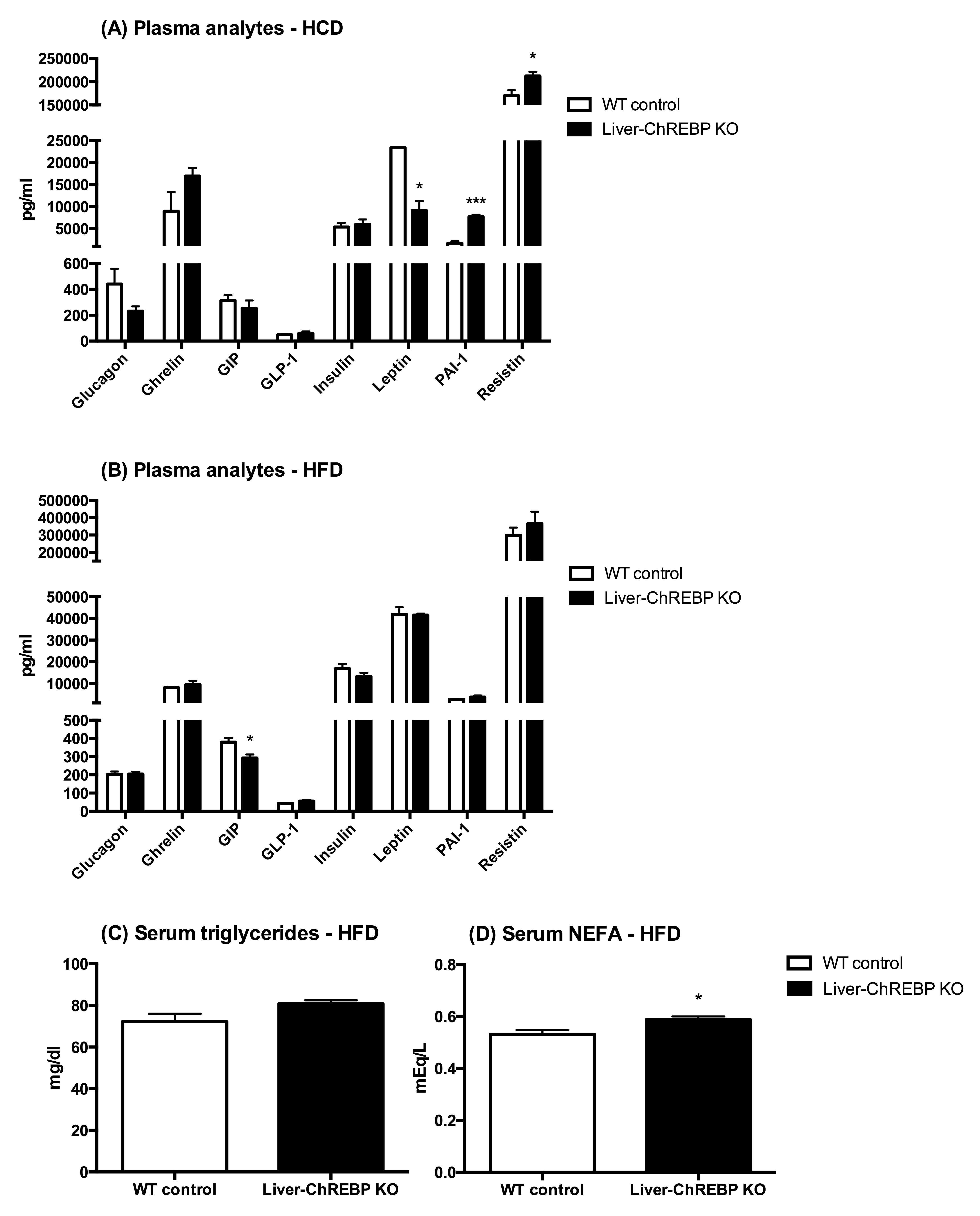


**Supplemental Figure 15. Plasma metabolic biomarkers in Liver-ChREBP KO mice under different dietary stressors**Concentrations of metabolic biomarkers in plasma of Liver-ChREBP KO and WT mice in the nonfasted state after **A.** 12 weeks HCD feeding (n=4 per group), or **B.** 12 weeks HFD feeding (n=5-6 per group).
**C.** Serum triglyceride and **D.** non-esterified fatty acid (NEFA) levels in Liver-ChREBP KO and WT mice fed a HFD for 12 weeks (n=5-6 per group).
Results expressed as mean ± SEM. Statistical analysis by unpaired t-test between each genotype (*: p<0.05, ***: p<0.001).
