# Supplementary material for: Deletion of hepatic carbohydrate response element binding protein (ChREBP) impairs glucose homeostasis and hepatic insulin sensitivity in mice
Source: Mol Metab. 2017 Jul 18;6(11):1381–94. doi: 10.1016/j.molmet.2017.07.006 (PMC5681238; doi:10.1016/j.molmet.2017.07.006)
Supplement: mmc2 [file mmc2.docx]

**ChREBP model methods**

ChREBP (Mlxipl) is a glucose-responsive basic helix-loop-helix leucine zipper transcription factor that is present in multiple cell types, but its regulatory function on cell metabolism is thought to be especially important in the liver, in which glucose controls ChREBP functions via multiple mechanisms including increased expression and transactivation activity, phosphorylation-dependent upregulation of nuclear translocation and DNA binding. To better determine the role of ChREBP in the liver, we have produced a liver specific ChREBP deficient mouse model.

We purchased a bacterial artificial chromosome (BAC) clone containing the mouse ChREBP gene from Invitrogen (Carlsbad, CA, USA). Two DNA fragments, 3.7 and 4.4 kb, were sub-cloned from this BAC by recombineering [1] and used for homologous recombination. A 0.5 kb DNA fragment containing the targeted exon 1 with its immediate 5′ and 3′ introns (partial) including the translational start codon of ChREBP was amplified by PCR and inserted in between two loxP sites of the NeoFrtLoxP vector. Two TK cassettes were inserted into the 5′-end of the targeting vector (Figure 1). We electrophorated R1 mouse embryonic stem cells [2] with a linearized targeting construct, and selected embryonic stem cells with G418 (Invitrogen) and ganciclovir. Blastocyst injection and germline transmission were done by standard techniques. After germ line transmission was confirmed by Southern blot analysis, ChREBP-targeted mice were bred into C57BL/6J mice for ≥6 generations. We identified floxed mice genotype by PCR analysis of tail DNA (Figure 2).

To generate liver ChREBP deficient mice, we bred ChREBP fl/fl mice with Albumin-Cre transgenic mice (AlbCre+/0). Cross-breeding of these mice produced liver specific homozygous deletion of ChREBP.

References:

1. Lee EC, Yu D, Martinez DV, et al. A highly efficient Escherichia coli-based chromosome engineering system adapted for recombinogenic targeting and subcloning of BAC DNA. Genomics. 2001; 73:56–65.
2. Nagy A, Rossant J, Nagy R, Bramow-Newerly W, Roder JC. Derivation of completely cell culturederived mice from early-passage embryonic stem cells. Proc Natl Acad Sci USA. 1993; 90:8424– 8428.


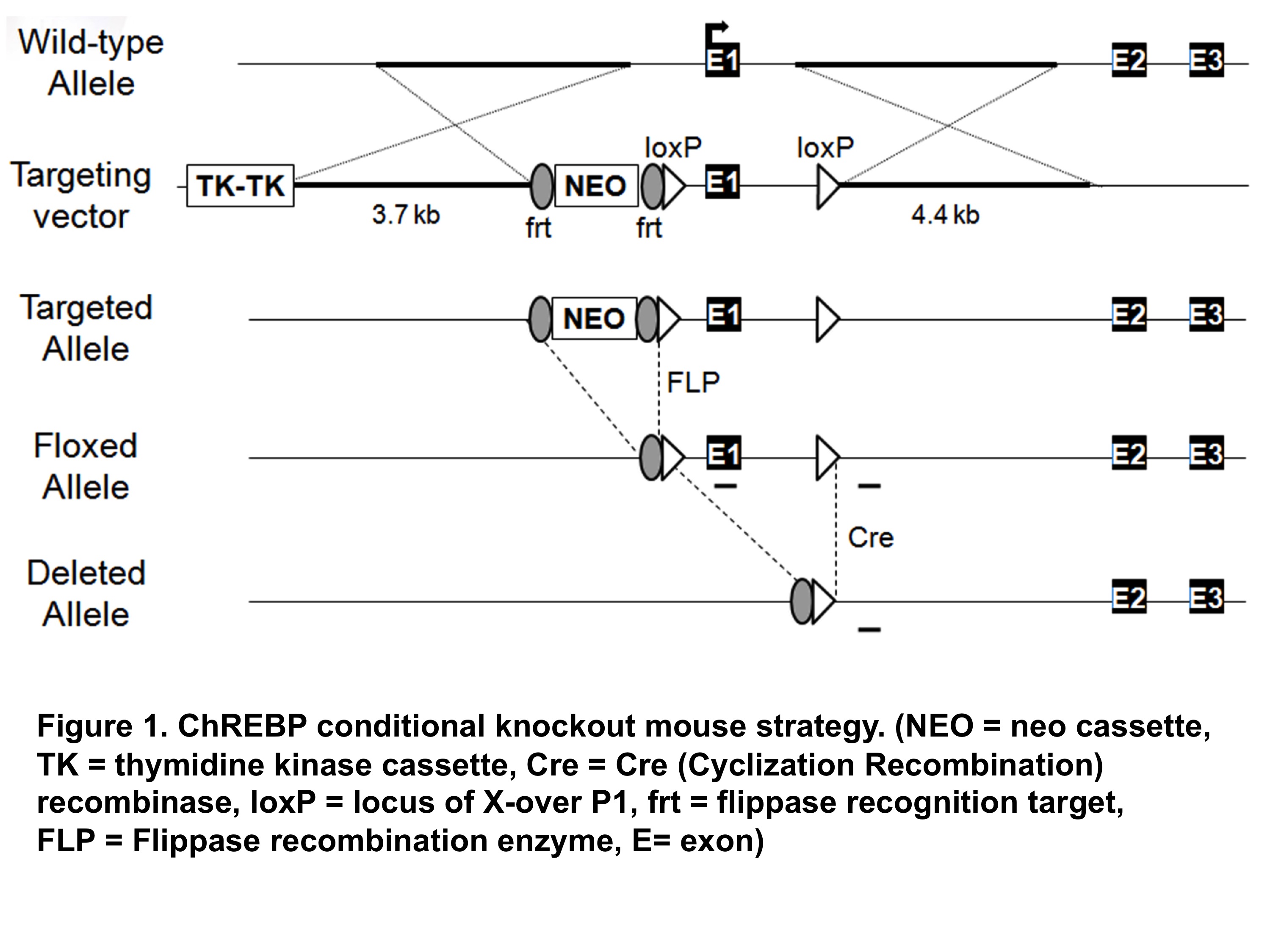


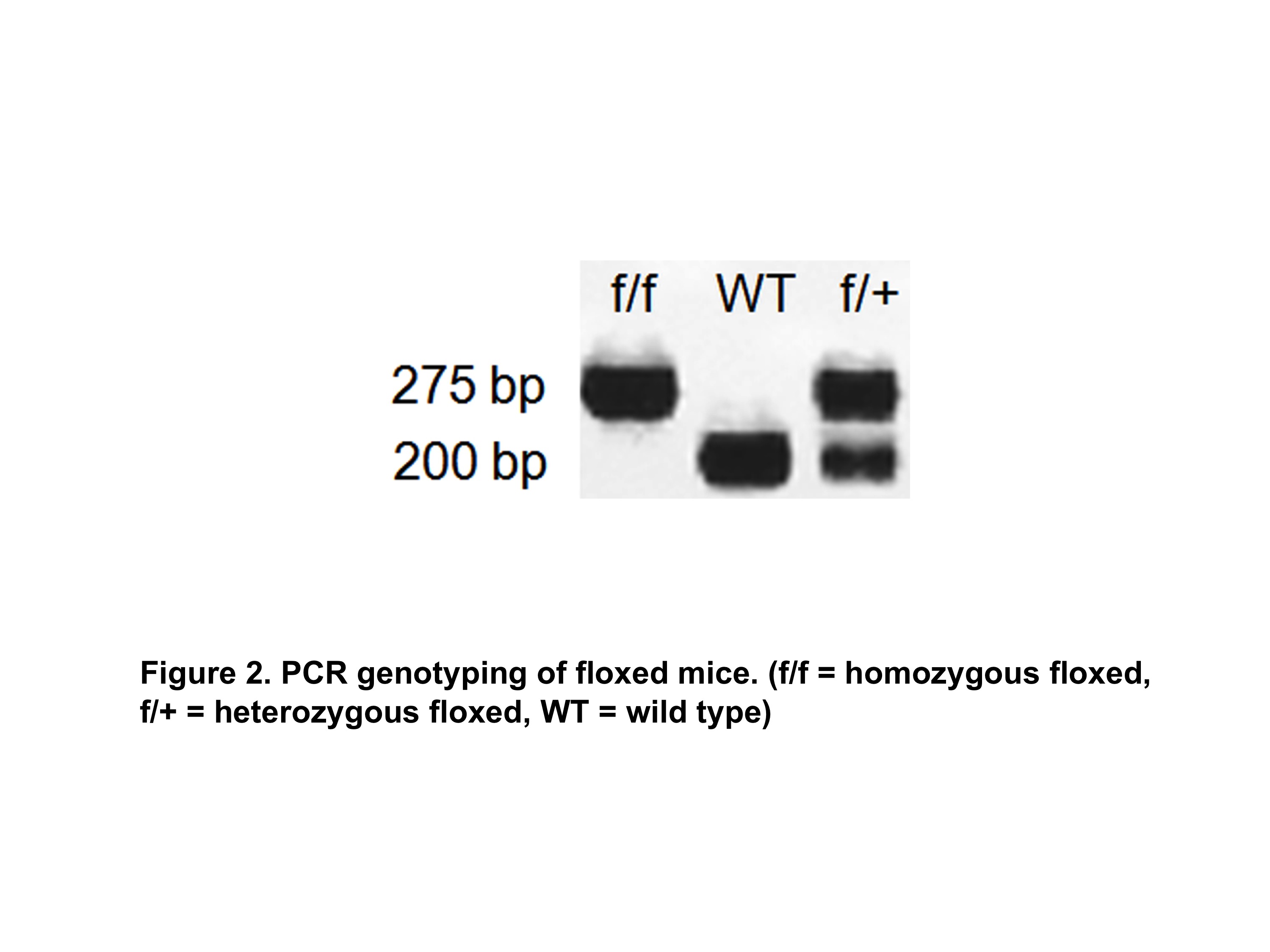


**AlbCre/Chrebp genotyping**

**Primers sequence:**

AlbCre forward primers: Cre51: CTGCATTACCGGTCGATGCA

AlbCre reverse primers: Cre31: CAGCATTGCTGTCACTTGGT

Expected band size for Cre positive pups: 300 bp

Chrebp forward primers: Chrebpgeno2-51: acccacctcttcgagtgct

Chrebp reverse primers: Chrebpgeno2-31: caagcaagtgatgggacaca

Expected band size: fl/fl: 275 bp, WT: 200 bp, fl/+: 275bp/200bp (both band)

**PCR procedure:**

step1: 94 ºC for 3 minutes

step2: 94 ºC for 30 seconds

step3: 58 ºC for 30 seconds

step4: 72 ºC for 30 seconds

step5: Goto step2, 32 times

step6: 72 ºC for 5 minutes

step7: hold at 4 ºC

| 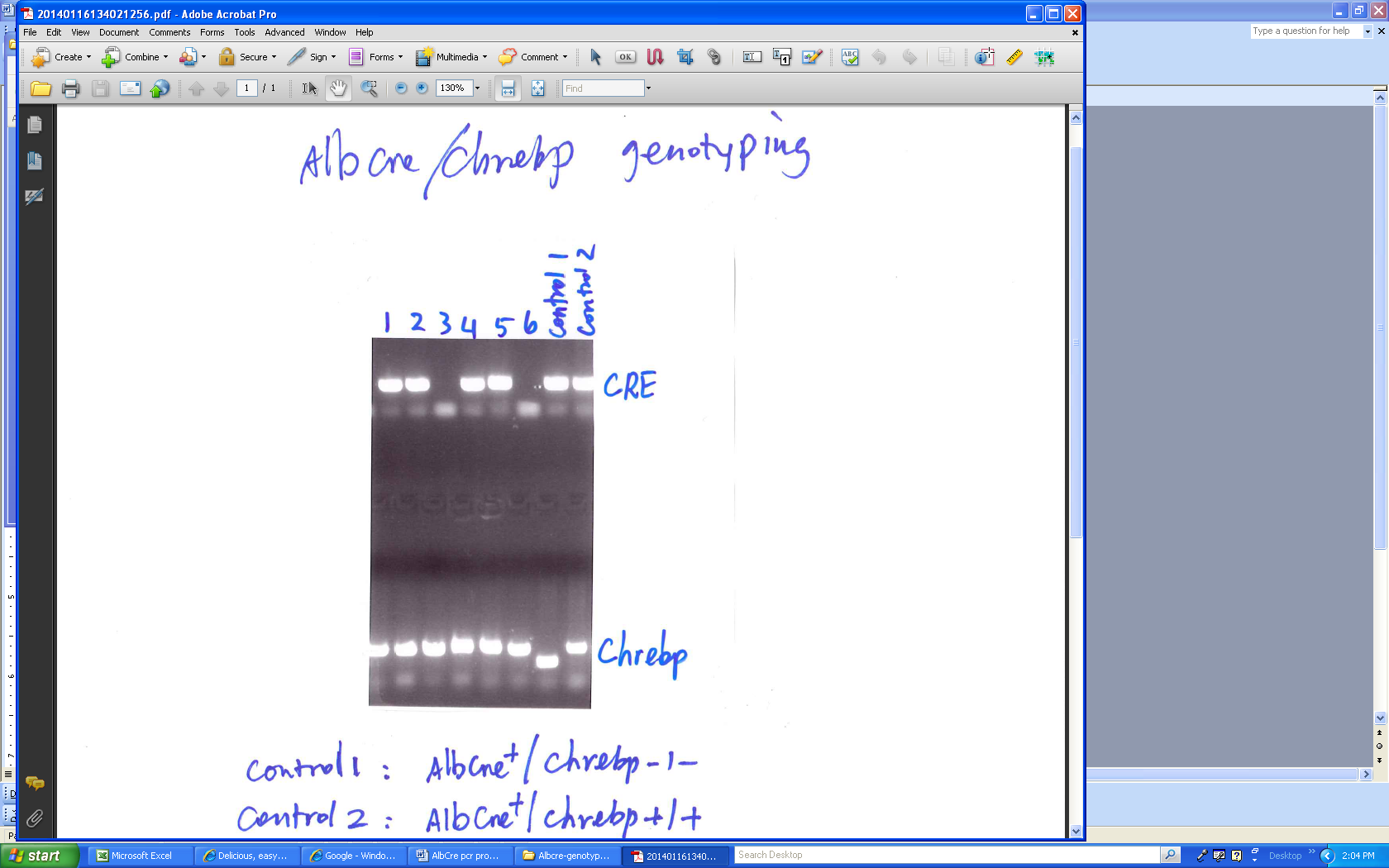  **275 bp**  **200 bp**  **300 bp** | Result:  Control1: AlbCre^+^/ Chrebp^+/+^  Control2: AlbCre^+^/ Chrebp ^fl/fl^  Sample1: AlbCre^+^/ Chrebp ^fl/fl^  Sample2: AlbCre^+^/ Chrebp ^fl/fl^  Sample3: AlbCre^-^/ Chrebp ^fl/fl^  Sample4: AlbCre^+^/ Chrebp ^fl/fl^  Sample5: AlbCre^+^/ Chrebp ^fl/fl^  Sample6: AlbCre^-^/ Chrebp ^fl/fl^ |
| --- | --- |
